# Machine learning frameworks to accurately estimate the adsorption of organic materials onto resin and biochar

**DOI:** 10.1038/s41598-025-99759-2

**Published:** 2025-04-30

**Authors:** Raouf Hassan, Mohammad Reza Kazemi

**Affiliations:** 1 Civil Engineering Department, College of Engineering, Imam Mohammad Ibn Saud Islamic University (IMSIU), Riyadh, 11432 Saudi Arabia; 2 Process Engineering Department, Bandar Imam Petrochemical Company (BIPC), Mahshahr, Iran

**Keywords:** Machine learning, Adsorption prediction, Biochar and resins, Monte Carlo outlier detection, SHAP analysis, Biochemistry, Chemistry, Energy science and technology, Engineering, Materials science

## Abstract

Adsorption prediction of organic components on biochar and resins is essential for advancing industrial and energy technologies. This study utilized a dataset of 1750 adsorption isotherms comprising adsorption data for 73 organic materials on 50 biochar samples and 30 polymer resins. Machine learning models were developed using eight input parameters, including five Abraham solvation descriptors, total pore volume (Vt), specific surface area (BET), and equilibrium concentration (logCe), with the output parameter being adsorption degree (logKd). The dataset was split into training (1225 data points), testing (262), and validation (263). Various machine learning methods were evaluated, including Linear Regression, Ridge Regression, Lasso Regression, Elastic Net, Support Vector Regression (SVR), k-Nearest Neighbors (KNN), Decision Trees, Random Forests, Gradient Boosting Machines, Artificial Neural Networks (ANNs), Convolutional Neural Networks (CNNs), Gaussian Processes, as well as ensemble algorithms such as XGBoost, LightGBM, and CatBoost. Among these, XGBoost achieved superior accuracy with an R² of 0.974 and a mean squared error (MSE) of 0.0343, followed by LightGBM (R²=0.964, MSE = 0.0484) and CatBoost (R²=0.984, MSE = 0.0212). Simpler models such as Linear Regression and Elastic Net showed lower performance, with R² values ranging from 0.678 to 0.875 and higher MSE values. Sensitivity and SHAP analyses identified equilibrium concentration and specific surface area as the most critical factors influencing adsorption. The findings underscore the effectiveness of machine learning methods, particularly XGBoost, LightGBM, and CatBoost, in forecasting adsorption levels with high precision while offering actionable insights into key variables driving adsorption mechanisms.

## Introduction

One of the most pressing issues impeding the progress of modern society is the profound deficit of safe and clean water^1,2^. Water resources and environmental integrity are increasingly endangered by organic pollutants, particularly those originating from personal care products, pesticides, and food additives^3^. Physical adsorption is an effective method for removing organic pollutants when considering water restoration efforts, primarily because of its economical nature, straightforward application, and minimal energy consumption^4–6^. Research has highlighted the effectiveness of multiple adsorbents, such as granular activated carbons, carbon nanotubes, biochars, and graphene nanosheets, in addressing the issue of organic pollutant removal from water sources^7–9^. Despite its potential, the inefficiency of currently utilized technologies, particularly in dealing with new chemical pollutants and modern adsorbents, has contributed to a critical shortage of reliable data on compound adsorption mechanisms^10–13^. Researchers can leverage existing data more effectively by creating accurate predictive frameworks, thereby minimizing the need for extensive and laborious adsorption experiments^14–16^. Frequently employed in adsorption studies, the polyparameter linear free energy relationship (pp-LFER) model is a reliable tool for estimating the adsorption tendencies of organic compounds^17^. The pp-LFER model elucidates the phase spreading of organic pollutants through its molecular structure parameters, which are designed to reflect the influence of intermolecular forces on adsorption processes^18^. The pp-LFER model’s coefficients provide valuable insights into the differences in intermolecular forces across various systems, aiding in understanding organic compound adsorption mechanisms and enabling reliable environmental risk assessments. However, the model’s tendency to disregard adsorbent properties often introduces considerable prediction errors. Moreover, its reliance on multilinear regression (MLR) for each equilibrium concentration (Ce) restricts its predictive scope to the concentration ranges utilized in the model’s development^14^. To address the shortcomings of existing methods, machine learning-based models that account for the intrinsic properties of adsorbents and organic compounds can serve as an effective and advanced solution.

As an interdisciplinary domain intersecting multiple disciplines, researchers now actively utilize machine learning (ML) in adsorption studies^19^. Machine learning (ML) is fundamentally centered on creating and examining algorithms that facilitate automated “learning” in computers. Grounded in statistical methodologies, these algorithms can dissect the architecture of existing data, uncover hidden trends, and employ this information to make informed predictions or decisions regarding new, unobserved data points^20^. ML methods, such as SVM, ANN, and RF, have steadily gained prominence as critical approaches in the investigation of organic pollutant adsorption processes^21^. Machine learning (ML) techniques offer the dual advantage of optimizing adsorption parameters^22,23^ and outperforming traditional regression methods in simulating adsorption behavior, whether for single-component or multi-component systems^24,25^.

ML approaches, including ANN, SVM, RF, and similar methodologies, have emerged as pivotal tools in the study of organic pollutant adsorption^21^. These ML techniques serve dual roles: they optimize adsorption parameters^22,23^ and outperform traditional regression approaches in modeling single-component and multi-component adsorption scenarios^24,25^. The ANN model has been utilized to explore biochar’s potential for predicting pollutant adsorption. Although the ANN algorithm yielded acceptable RMSE and R² values, some aspects of its predictive accuracy were limited^26^. Furthermore, LS-SVM and ANN have been validated for improving the adsorption efficiency of methylene blue fuel^27^. A comparative analysis of feedforward backpropagation ANN and RF models was performed to evaluate their effectiveness and suitability for predicting energy consumption in 336 instances. Both models demonstrated robust performance during the training and validation stages^28^. However, no comprehensive model to predict the adsorption of organic materials on many biochar and polymer resins is available, which is the focus of this work.

**Fig. 1:**
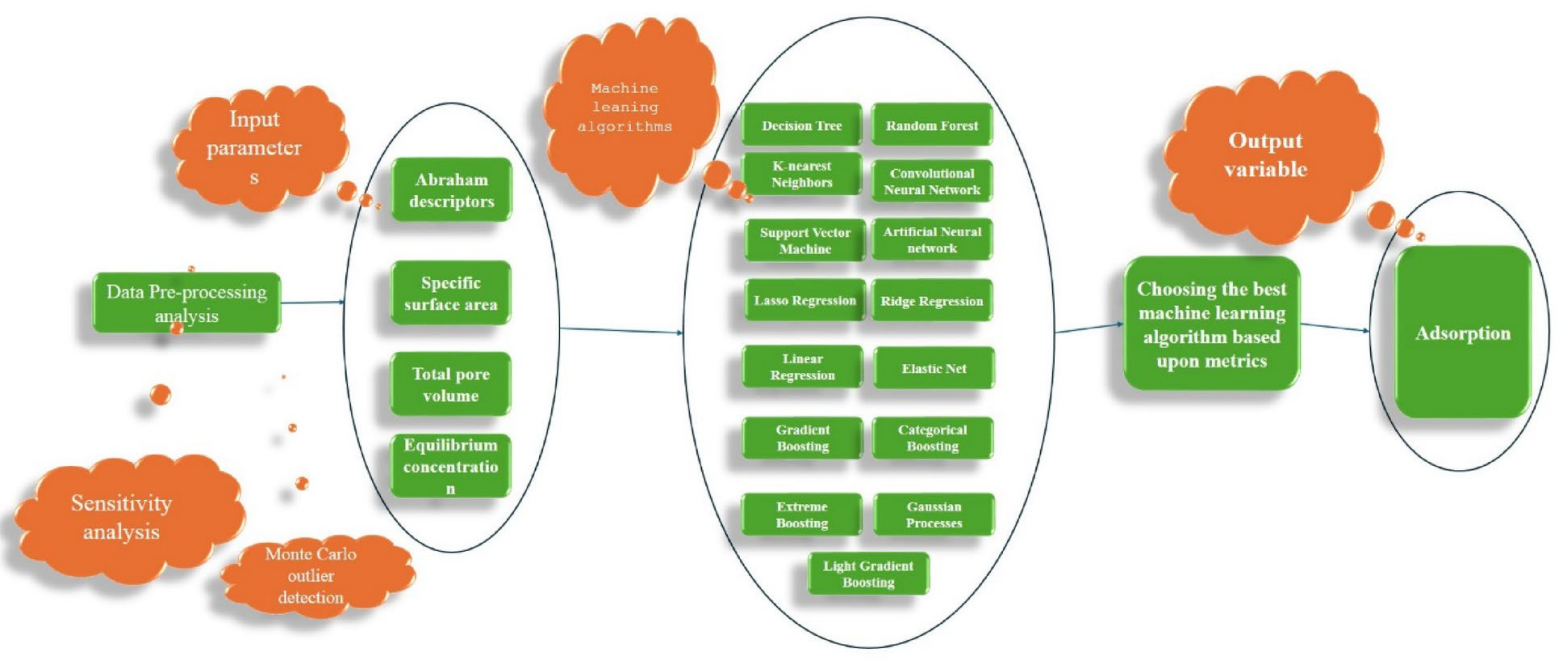
Summary of the workflow used to compute adsorption values of organic materials onto biochar and polymer resins.

This research plans to make machine learning data-driven models that compute the adsorption of organic materials on biochar and polymer resins. A broad spectrum of ML techniques, such as linear regression, convolutional neural networks, artificial neural networks, ridge regression, elastic net, lasso regression, SVM, random forests, GBM, KNN, decision trees, extreme boosting, light gradient boosting, categorical boosting, and Gaussian processes, is employed to model adsorption mechanisms. The Monte Carlo outlier detection algorithm is applied to ensure the dataset’s robustness for training purposes. The models are rigorously evaluated using various performance indicators and graphical analyses. Additionally, SHAP values are analyzed for the most effective model to elucidate the role of key features in influencing adsorption data. A detailed representation of the methodological approach is given in Fig. 1.

This paper is outlined into several sections. First, the background of the utilized machine learning algorithms is given. Then, the collected dataset is analyzed in terms of sensitivity analysis, specification of input parameters for the data-driven modeling, and outlier detection using Monte Carlo algorithm. Then the developed models are assessed according to their unveiled evaluation metrics, the top-performing of which is determined ultimately. Finally, the future work with regard to this paper is illuminated in detail. This study indeed builds upon a comparative study in which the efficiency of several machine learning algorithms is performed in order to find out the top-performing one for the prediction of adsorption values of organic materials onto biochar and polymer resins.

## Machine learning backgrounds

This section explains each of the implemented machine-learning algorithms.

### Convolutional neural network

**Fig. 2:**
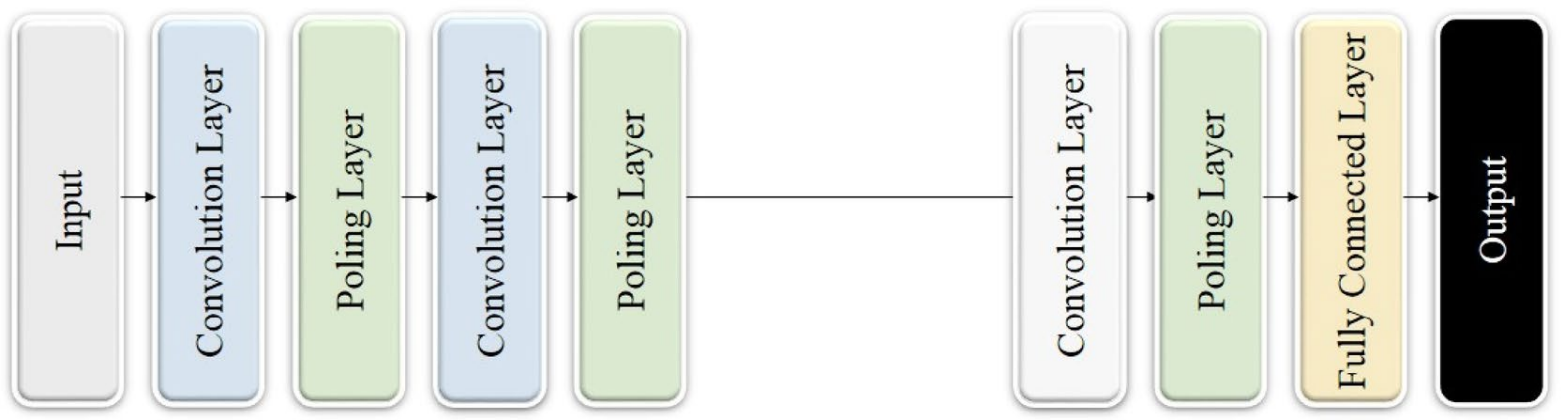
Architecture of the convolutional neural networks applied in this study.

Figure 2 demonstrates that CNNs represent a novel type of deep learning model crafted to efficiently handle grid-like structured data, rendering them particularly suitable for tasks involving images and spatial data^29^. The main characteristics of CNNs, such as convolutional layers, pooling techniques, and hierarchical feature extraction, enable the model to automatically and adaptively learn intricate spatial hierarchies of features^30,31^. This has greatly impacted many fields, including computer vision, pattern recognition, and multi-dimensional signal processing.

CNNs have revolutionized the deep learning field due to their remarkable ability to progressively extract more complex and abstract features directly from raw data, such as images. This is achieved using two essential architectural ideas: local connectivity designs and common parameters.

Enhancements in CNN frameworks have greatly expanded the possibilities of deep learning, addressing critical issues related to computational complexity, interpretability, and generalization across various domains^55^. In the past few years, particularly since 2020, research has focused on developing new network architectures, including lightweight CNNs, hybrid models based on transformers, and self-supervised learning methods that lessen the dependence on large labeled datasets^32,33^. Recent advancements in Convolutional Neural Networks (CNNs), such as neural architecture search, attention mechanisms, and improved regularization methods, have significantly improved their effectiveness in tackling complex real-world challenges^34^.

### Artificial neural network

ANNs are neural networks inspired by the human brain, consisting of interconnected nodes representing input, hidden, and output layers. These networks process data by computing weighted sums of inputs, applying an activation function, and propagating the result to deeper layers. Mathematically, a neuron operates as follows:

1 $$h(x)=f\left( {\sum\limits_{{i=1}}^{n} {{\omega _i}{x_i}+b} } \right)$$

Where ω are weights, x are inputs, b is the bias term, and f is the activation function (e.g., sigmoid or ReLU). Optimization methods such as gradient descent minimize error during training.

ANNs are widely applied in image identification, natural language processing, and speech recognition. Techniques like dropout and transfer learning mitigate overfitting, ensuring adaptability to modern applications. Studies such as those by Zhu et al. and Heidari et al. highlight ANNs’ transformative role in deep learning, bridging computational advancements and applications^35,36^.

### Decision tree

**Fig. 3:**
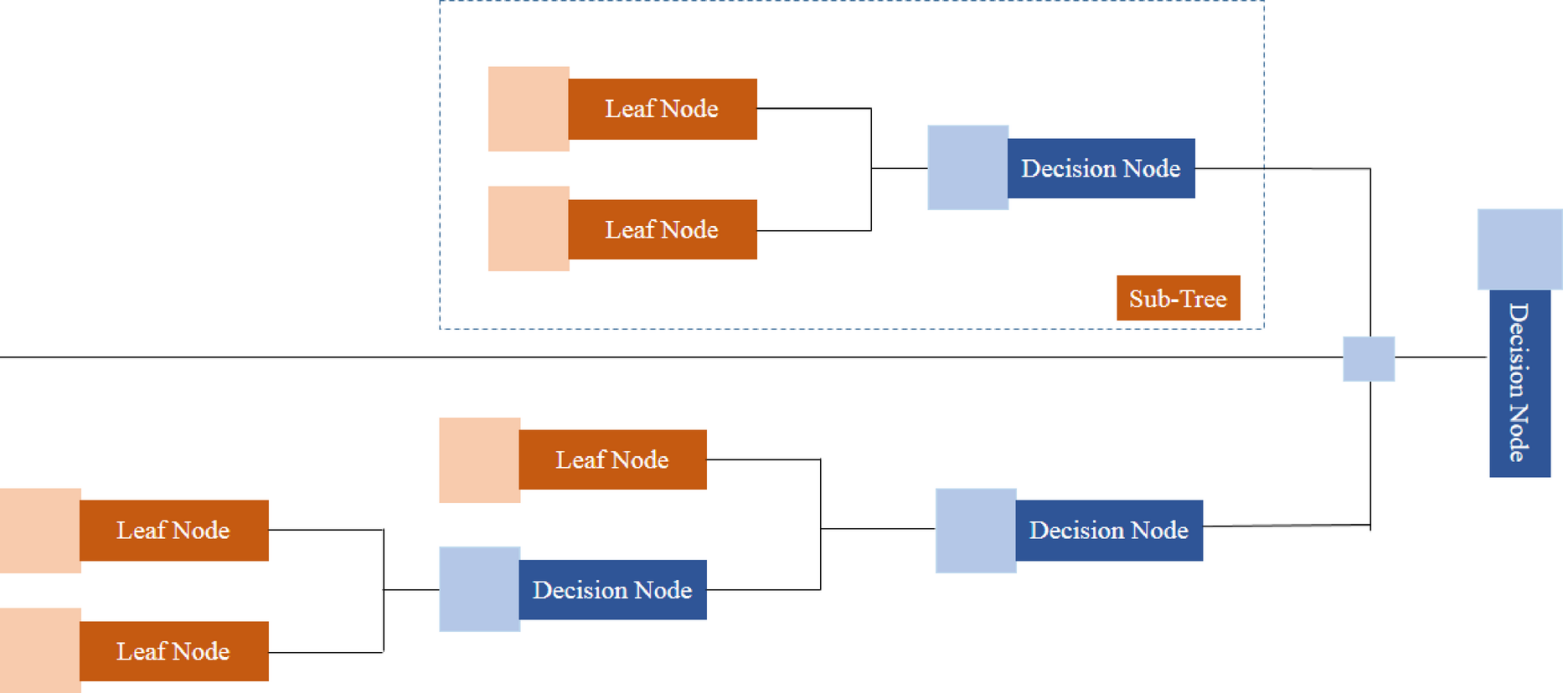
The structural layout of the decision tree algorithm.

As shown in Fig. 3, a decision tree is a layered configuration displaying a flowchart that aids decision-making by representing features as internal nodes, branches as decision rules, and outcomes as leaf nodes. The tree begins with a root node at the top, and data is divided recursively according to attribute values. This straightforward and comprehensible framework allows for easy visualization of the decision-making process, which is why decision trees are commonly preferred for classification or regression tasks in machine learning^37–40^.

Each node in the tree evaluates a specific attribute or factor, while the branches represent possible outcomes or selections. The tree grows by choosing the attribute and decision criterion that lowers impurity or improves information gain. In decision tree algorithms, the process described earlier involves the recursive splitting of the dataset based on the feature values. At every node, the algorithm selects a feature to split the dataset into two or more subsets, improving an objective function such as information gain, Gini index, or chi-squared test.

Decision trees can handle numerical and categorical data and are often used for selecting features, detecting outliers, and interpreting models. They may operate as standalone models or as parts of more complex ensemble methods, such as random forests or gradient boosting. The clarity of decision trees makes them attractive for situations where understanding the core decision-making process is crucial^41–43^. When building decision trees, metrics like Gain Ratio and Gini Index are essential for determining the optimal split to divide the data. These techniques assist in identifying the best feature and decision criterion at every node, thus enhancing the accuracy of the tree^44–47^.

### Random forest

**Fig. 4:**
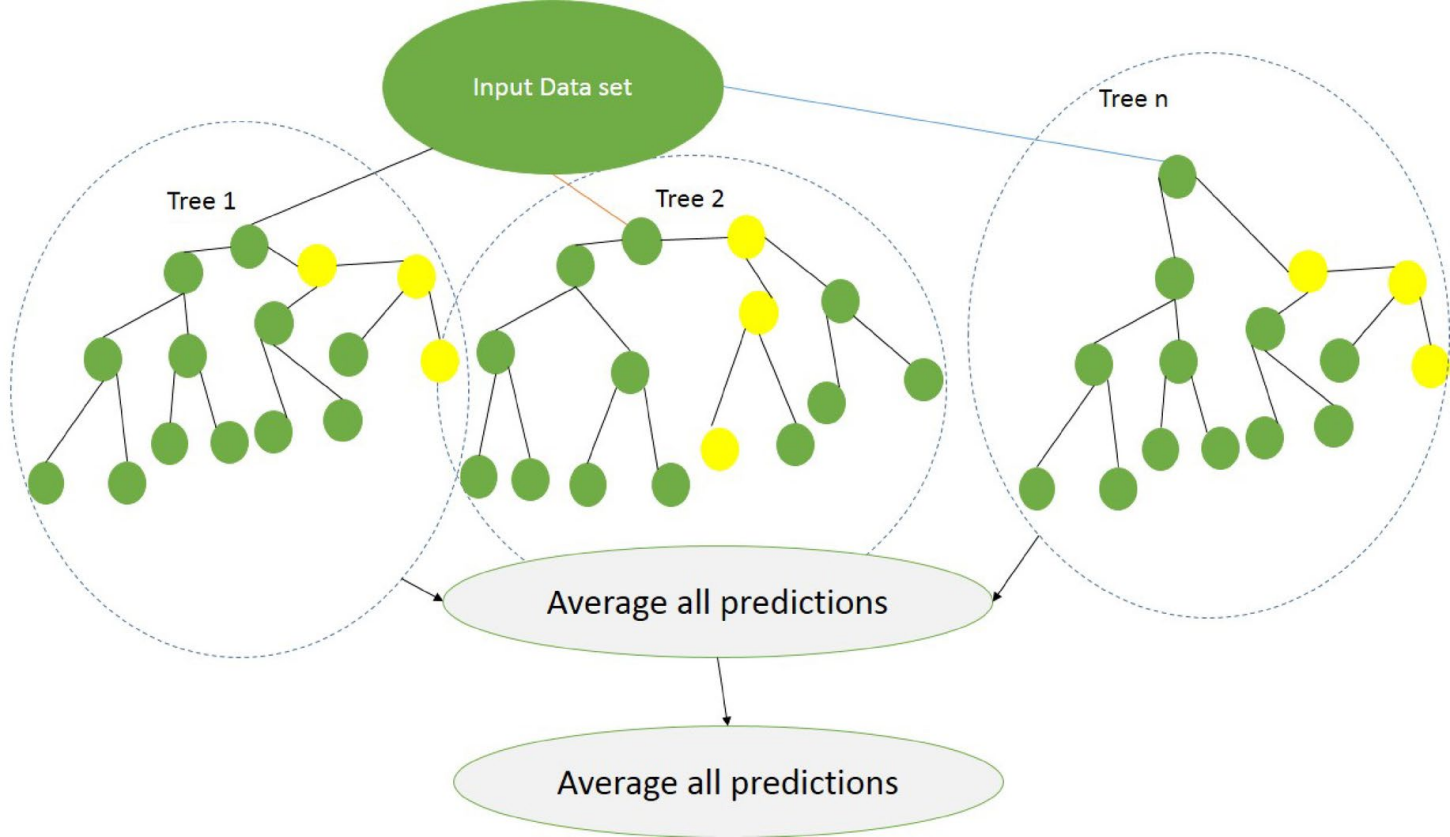
Schematic representation of the random forest algorithm.

As depicted in Fig. 4, Random Forest is a consisted of classifications and regressions made on datasets corresponding to the size of the training set, known as bootstraps, which are created by randomly resampling from the training portion of the dataset^48^. When a tree is made, a set of bootstraps that omits any individual entry from the initial dataset acts as the testing dataset. The categorization of error magnitudes across all testing sets indicates the out-of-bag evaluation of generalization errors. It has been shown before that for bagged classifiers. The out-of-bag error is as accurate as using a test set with the same data points as the training set. Consequently, employing the out-of-bag estimation removes the need for a separate testing set. To classify new input data points, each classification and regression tree votes for a class, and the forest determines the class that receives the highest number of votes^49^. The approach uses specific guidelines regarding tree construction, tree combination, self-assessment, and post-processing, showing strength against over-fitting.

In comparison to other machine learning methods, it is deemed to be more reliable when dealing with outliers or in very large-dimensional parameter spaces^50^. The concept of variable importance is an inherent feature selection performed by RF employing a random subspace method, assessed using the Gini impurity index criterion. The Gini index evaluates the strength of predictive variables for regression and classification based on minimizing impurities. To achieve the best split of a binary node, maximizing the improvement in the Gini index is essential. In basic terms, a low Gini suggests that a particular predictor^48,50,51^.

### Linear regression

The approach matches data on linear models, where the relation between y and independent x is modeled as:

2 $$y = \omega _{0} + \sum\limits_{{j = 1}}^{n} {\omega _{j} x_{j} + \varepsilon }$$

Ω_j_ is the weights, ω_0_ is the intercept, and ∈ is the error term. Despite simplicity, assumptions like linearity and homoscedasticity may not align with real-world data. Its relevance spans predictive analytics and remains a foundation for regression-based modeling, highlighted in works such as Chen et al.^52^.

### Ridge regression

The technique is an extension of linear regression that addresses multicollinearity and overfitting by adding an L2 regularization term to the loss function. This term penalizes the squared magnitude of the coefficients, which prevents them from growing too large and reduces the model’s sensitivity to slight variations in the data. By introducing this penalty, ridge regression ensures better generalization, particularly in high-dimensional datasets with highly correlated predictors. It balances the trade-off between fitting the data and maintaining model simplicity. Typical applications include finance, genomics, and machine learning, which are valuable for improving predictive accuracy in complex or noisy datasets with many features.

Ridge Regression extends linear regression by adding L2 regularization to the cost function:

3 $$L = \sum\limits_{{i = 1}}^{n} {(y_{i} - \hat{y}_{i} )^{2} + \lambda \sum\limits_{{j = 1}}^{p} {\omega _{j}^{2} } }$$

L is the loss function, λ controls regularization, and ω are coefficients. This penalization prevents coefficients from becoming too large and improves model stability in datasets with multicollinearity. Hoerl and Kennard’s work laid the foundation for Ridge Regression, establishing its effectiveness in high-dimensional datasets^53,54^.

### Lasso regression

This approach is a linearized regression variation that addresses overfitting and multicollinearity but focuses on feature selection. Unlike ridge regression, which uses an L2 penalty, lasso regression adds an L1 penalty to the loss function, which is proportional to the absolute value of the coefficients. This L1 penalty forces several coefficients to zero, excluding some features. This characteristic makes lasso regression particularly useful for feature selection in high-dimensional datasets, where many predictors may be irrelevant or redundant. Shrinking some coefficients to zero helps improve model generalization and provides a simpler, more interpretable model by identifying the most important predictors. Lasso regression is ideal when it has many features and wants to reduce the complexity of the model by eliminating those that do not contribute meaningfully to the prediction. The result is a sparse model that is easier to interpret and less prone to overfitting, particularly in cases with many correlated predictors^55,56^.

Applications of Lasso Regression are diverse and span multiple domains. In genomics, it is used to select a subset of genes that contribute most significantly to a disease outcome, effectively decreasing the dimensionality of the data while bringing the most crucial predictors. In finance, lasso regression can help identify key factors influencing asset prices or credit risks, especially when the number of potential predictors (such as economic indicators or market metrics) is significant. In marketing, it is applied to determine which channels or features (e.g., pricing, advertising, or product attributes) most impact consumer behavior, allowing businesses to allocate resources efficiently. Furthermore, in machine learning, lasso regression is commonly used for sparse modeling in high-dimensional datasets, such as image processing or text classification, where only a small subset of features might be relevant for accurate predictions. Its ability to perform feature selection while preventing overfitting makes it especially useful in applications involving large-scale, complex datasets.

Lasso Regression adds L1 regularization to the cost function:

4 $$L = \sum\limits_{{i = 1}}^{n} {(y_{i} - \hat{y}_{i} )^{2} + \lambda \sum\limits_{{j = 1}}^{p} {|\omega _{j} |} }$$

This penalty shrinks some coefficients to zero, effectively enabling feature selection. Lasso is particularly helpful for high-dimensional data, where irrelevant predictors must be excluded. Zhang et al. and Zhan et al. work on Lasso Regression underscores its utility in sparse modeling^57,58^.

### Support vector regression

SVR is a regression approach that uses the principles of SVM, typically used for classification tasks but adapted to handle regression problems. In SVR, the model aims to find a function approximating the underlying relationship between input and output variables. However, with an important distinction: it does not aim to minimize the error directly for all points but instead focuses on finding a balance between error tolerance and model complexity. SVR defines a tolerance margin, represented by a “tube” (or epsilon-insensitive tube), within which errors are not penalized. Data points within this margin are considered well-predicted, while data points outside the margin incur a penalty based on how far they lie from the predicted values^59,60^.

SVR can be implemented in both linear and nonlinear forms. In the nonlinear case, SVR employs the kernel trick, which maps the input data into a higher-dimensional space, allowing the model to capture complex, nonlinear relationships. Popular kernels include the Radial Basis Function (RBF) kernel, which helps capture highly complex, nonlinear patterns in the data. VR is powerful when dealing with noisy data or when we need a model that balances fitting the data with avoiding overfitting. It is often used in financial forecasting, engineering, and time-series analysis, where precision and robustness against outliers are important. Its ability to work well with high-dimensional data and its inherent regularization properties make it a valuable tool in many fields requiring predictive modeling^47^.

### Gradient boosting machine

GBM, introduced by Jerome Friedman in 1999, is a supervised ensemble learning method that builds a robust predictive model by combining multiple decision trees. This iterative approach aims to reduce the errors of the existing trees and enhance a loss function to increase accuracy in classification and regression tasks^61,62^. GBM is particularly valued for its ability to handle complex, nonlinear connections and its capability to provide insights into the importance of features and aid in feature selection. However, despite these advantages, GBM can be demanding regarding resources and needs careful tuning of hyperparameters, such as the learning rate and depth of trees, to balance the trade-offs between overfitting and underfitting.

The algorithm uses a step-by-step, iterative approach, beginning with applying a simple model, such as a decision tree, to the data. The primary objective is to minimize the loss function L(y, f(x)), where y represents the true target, f(x) denotes the predictions generated by the model, and L is the loss function. The model is gradually refined at each stage to reduce mistakes and improve its predictions^63,64^.

The initial phase, which constitutes the first iteration, is outlined as follows.

5 $$F_{0} (x) = \mathop {\arg \min }\limits_{c} \sum\limits_{{i = 1}}^{n} {L(y_{i} ,C)}$$

The second stage, for every iteration m, is as follows:

Calculate the negative gradient (pseudo-residuals):

6 $$r_{{im}} = - \left[ {\frac{{\partial L(y_{i} ,F(x_{i} ))}}{{\partial F(x_{i} )}}} \right]_{{F(x) = F_{{m - 1}} (x)}}$$

The next stage: Fit a base learner to these residuals:

7 $$h_{m} (x) = \mathop {\arg \min }\limits_{h} \sum\limits_{{i = 1}}^{n} {(r_{{im}} - h(x_{i} ))^{2} }$$

Fourth stage: Update the model:

8 $$F_{m} (x) = F_{{m - 1}} (x) + \upsilon .h_{m} (x)$$

**Fig. 5:**
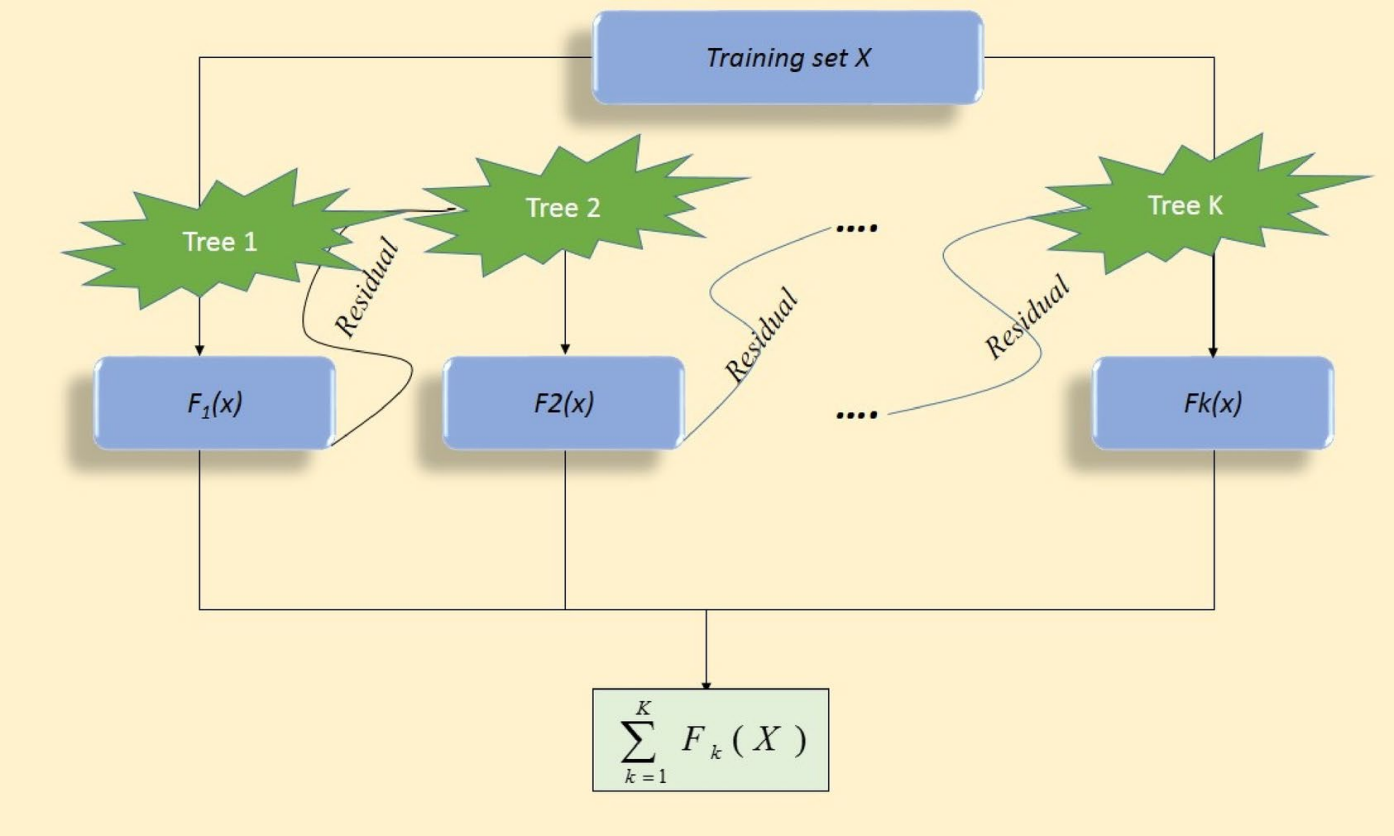
Schematic flow of the gradient boosting machine (GBM) algorithm.

In the GBM algorithm, the learning rate, υ determines how much each decision tree impacts the overall model. In each iteration, the algorithm adds a new decision tree to correct the errors in the combined predictions of all previous trees. The flexibility of GBM arises from its ability to customize the loss function (L) to achieve specific objectives, making it an extremely adaptable approach. Regularization parameters such as the learning rate (υ) and depth of the tree are included to enhance model generalization and prevent overfitting. As a result, the final classifier is a weighted sum of predictions from individual trees, gradually grasping more complex patterns in the data^65–67^. A diagrammatic illustration of the GBM algorithm is shown in Fig. 5.

###  K-nearest neighbors

KNN is mainly utilized in classifications and regressions. For classifications, the algorithm calculates the difference between the query point and all points in the training set using metrics like Euclidean, Manhattan, or Minkowski distance. It then identifies the K’s nearest neighbors and assigns the class that appears most frequently among them. In regression, KNN predicts the target value by averaging (or weighting) the values of the K nearest neighbors. The choice of K and the weighting scheme significantly influence model performance. Importantly, KNN is computationally intensive since it requires storing the entire dataset and performing distance calculations at runtime, making it less efficient with large datasets. Despite this, KNN is valued for its simplicity and effectiveness, particularly when the data is well-distributed and no strong assumptions are made about its underlying distribution.

KNN has a wide range of applications across various fields due to its versatility and ease of implementation. In medicine, it is used for disease diagnosis, classifying patients based on features such as age, medical history, and test results. In finance, it aids in credit scoring and fraud detection by classifying transactions or customers as safe or suspicious. In e-commerce, KNN is applied in customer segmentation and personalized recommendations by analyzing customer behaviors. The algorithm is also valuable in image recognition and computer vision for tasks like facial recognition and object detection, where it classifies images based on pixel features.

Additionally, in geospatial analysis, KNN is used to predict geographic features, cluster regions with similar characteristics, and analyze land use patterns in urban planning and agriculture areas. The algorithm performs exceptionally well in problems with irregular decision boundaries and high-dimensional data settings where similar data points cluster closely in the feature space^47,68^.

### Extreme gradient boosting

The technique is an efficient ML based on GBM principles, primarily used for classification and regression tasks. It starts with a base model, usually a simple decision tree, and iteratively builds additional trees to eliminate the errors. This correction is done by focusing on the residuals—i.e., the differences between the predicted and actual values of the previous models. Each subsequent tree is trained using gradient descent to minimize these residuals, adjusting the model’s predictions accordingly. One of the algorithm’s strengths is incorporating L1 and L2 regularization (Lasso and Ridge) to prevent overfitting, promoting more straightforward and generalizable trees. Another key feature is its use of weighted quantile sketching, which handles sparse data efficiently. Early stopping is also employed to prevent overfitting by halting the training when the model’s performance on a validation set starts to degrade. The final model prediction is obtained by aggregating the outputs of all trees, where the contribution of each tree is weighted according to its performance. XGBoost also allows parallelization, making it faster than other gradient-boosting algorithms. The flexibility to tune hyperparameters like learning rate, max depth, subsampling ratio, and number of trees further enhances its performance and computational efficiency. These attributes make it particularly effective in handling large datasets and complex data patterns, leading to its widespread use in machine learning competitions and production environments.

XGBoost’s versatility makes it highly effective across various industries. It is widely used in finance for credit scoring, risk assessment, and fraud detection. It builds predictive models that estimate the likelihood of events such as loan defaults or fraudulent transactions. In healthcare, XGBoost aids in disease prediction and medical image classification thanks to its capacity to capture intricate patterns in high-dimensional medical data. In e-commerce, it is utilized for customer segmentation, recommendation systems, and demand forecasting, efficiently handling vast customer interaction datasets. Marketing applications also benefit from using XGBoost to predict customer behavior, optimize ad targeting, and improve sales forecasting. Due to its robustness in managing sparse textual data, the algorithm is valuable in Natural Language Processing (NLP) tasks, such as text classification, sentiment analysis, and spam detection. Energy forecasting, image recognition (including autonomous driving and facial recognition), and geospatial analysis for predicting geographic trends have significantly improved with XGBoost’s predictive capabilities. Its success in machine learning competitions highlights its ability to solve diverse and complex prediction and classification problems across industries, solidifying its position as a go-to tool for machine learning practitioners^69,70^.

### Gaussian process machine

The approach is a powerful Bayesian machine-learning model for regression and classification tasks. It models the relationship between inputs and outputs as a collection of random variables, where any subset follows a Gaussian distribution. Mathematically, GP assumes that the function f(x) generating the predictions is distributed as:

9 $$f(x) \sim GP(m(x),k(x,x\prime ))$$

m(x) denotes the mean function and k(x, x′) presents the covariance kernel defining the relationship between inputs x and x′. The kernel k(x, x′) is critical and can take forms such as the RBF (Radial Basis Function) kernel:

10 $$k(x,x\prime ) = \exp \left( {\frac{{|x - x\prime |^{2} }}{{2\ell ^{2} }}} \right)$$

where ℓ is the length scale parameter.

GPs provide uncertainty estimates for predictions, making them highly suitable for robotics, geostatistics, and optimization applications. However, GP models struggle with scalability as their computational complexity grows cubically with the number of data points. Techniques such as sparse approximations help mitigate this challenge. Wenming et al. and Su et al. provide a pivotal reference for Gaussian Processes, explaining their theory, applications, and kernel design^71,72^.

### Light gradient boosting machine

The technique is an efficient, scalable gradient-boosting framework developed by Microsoft that is designed to handle large datasets with high dimensionality while optimizing speed and performance like traditional gradient-boosting methods, building various decision trees, where each new tree corrects the errors made by the previous one. However, LightGBM incorporates several optimizations to enhance its performance, most notably its leaf-wise tree growth strategy, as opposed to the level-wise approach used by other boosting methods. This allows the model to converge faster and achieve better accuracy by focusing on the most impactful data splits. Additionally, LightGBM uses histogram-based algorithms that reduce both computational time and memory usage, making it suitable for large-scale datasets. Its advanced features, including categorical feature handling, parallel and GPU learning support, and built-in cross-validation, make it a powerful tool for rapid model deployment and real-time predictions.

LightGBM is extensively used across various industries for its speed, efficiency, and ability to handle vast data. It powers applications such as credit scoring, risk assessment, fraud detection, and high-frequency trading in finance. It supports predictive modeling for patient outcomes, treatment effectiveness, and disease diagnosis in healthcare. Its rapid computation and accuracy make it ideal for e-commerce and marketing tasks, including recommendation systems, customer segmentation, and churn prediction. LightGBM optimizes network operations and service quality in telecommunications and technology through complex data analysis. The algorithm is also widely applied in NLP tasks like text classification and sentiment analysis, leveraging its ability to handle sparse data. LightGBM’s versatility extends to forecasting applications, such as energy consumption prediction and sales forecasting, underscoring its broad utility in real-time data analysis and decision-making processes. Its exceptional performance and ease of use make it a preferred tool for machine learning practitioners seeking efficient and high-accuracy models^65,73^.

### Elastic net

Elastic Net is a regularization technique that combines the benefits of both Lasso (L1 regularization) and Ridge (L2 regularization) regression methods, making it highly effective for datasets with numerous predictors, significantly when some predictors are correlated or when the number of predictors exceeds the number of observations. Unlike Lasso, which sets some coefficients to zero for feature selection, and Ridge, which shrinks coefficients without eliminating them, Elastic Net combines L1 and L2 penalties. This hybrid approach mitigates the limitations of each method: Lasso’s tendency to select only one variable from correlated predictors and Ridge’s inability to eliminate any variables. By balancing both penalties, Elastic Net can perform feature selection while maintaining model stability, even in the presence of highly correlated predictors.

This technique is beneficial for high-dimensional datasets where multicollinearity is a concern. Lasso can result in a model where only one variable from a group of highly correlated features is retained, potentially missing important predictors. In contrast, Elastic Net retains multiple correlated features, enhancing model accuracy and interpretability. The technique’s flexibility comes from its key parameters: λ (lambda), which controls the regularization strength, and α (alpha), which determines the mix of Lasso and Ridge penalties. When α equals 1, Elastic Net behaves like Lasso; when α equals 0, it behaves like Ridge. Cross-validation is commonly used to find the optimal values of λ and α, ensuring that the model generalizes well to unseen data. Elastic Net’s ability to combine sparsity with stability makes it a robust feature selection and regularization solution, particularly in complex, high-dimensional datasets.

It helps address multicollinearity while performing feature selection for high-dimensional datasets. The objective function is expressed as:

11 $$L=\sum\limits_{{i=1}}^{n} {{{({y_i} - X_{i}^{T}\beta )}^2}+{\lambda _1}\sum\limits_{{j=1}}^{p} {|{\beta _j}|+{\lambda _2}\sum\limits_{{j=1}}^{p} {\beta _{j}^{2}} } }$$

Where λ_1_ controls the sparsity (lasso penalty) and λ_2_ controls shrinkage (ridge penalty). Elastic Net finds an optimal balance between feature selection and regularization, making it highly useful when predictors are highly correlated, or there are more features than data points.

Elastic Net is widely applied in domains like genomics, where selecting the most informative predictors among thousands of features is crucial. Guo et al. introduced Elastic Net, emphasizing its ability to overcome lasso’s limitations in multicollinear settings while preserving interpretability^74,75^.

### Categorical boosting

CatBoost is a high-performance gradient boosting library developed by Yandex that is designed to efficiently handle categorical features in data modeling tasks involving mixed feature types. Unlike traditional gradient boosting methods that require manual categorical data preprocessing, CatBoost automates this process through permutation-driven transformations and ordered boosting. These innovations lead to improved accuracy and reduced overfitting. The library also introduces symmetric trees for faster model inference, reduced prediction latency, and support for GPU acceleration, enabling efficient processing of large datasets. CatBoost’s robustness, ease of use, and native capability to process categorical data have made it popular in various applications, including finance and e-commerce. Its competitive performance frequently surpasses traditional gradient-boosting methods in modeling accuracy and computational efficiency, making it an invaluable tool for data scientists and analysts.

CatBoost’s efficient handling of categorical data makes it widely applicable across various industries. In finance, it aids in credit scoring, fraud detection, and risk analysis, leveraging categorical features like customer demographics and transaction types. In e-commerce, it enhances recommendation systems, personalized marketing, and customer segmentation by analyzing user preferences and behaviors, even with incomplete data. The library is also beneficial in healthcare for predicting patient outcomes and disease diagnosis using mixed data types such as medical records and test results. Additionally, CatBoost supports churn prediction, targeted advertising, and sales forecasting with rich customer datasets in marketing.

Furthermore, it is applied in natural language processing tasks like text classification and sentiment analysis, thanks to its capability to handle mixed categorical and text data. Its scalability and efficiency make CatBoost popular for real-time predictive analytics and large-scale modeling across retail, telecommunications, and gaming sectors.

CatBoost is a gradient-boosting algorithm specifically optimized for categorical features, relying on ordered target encoding and randomized permutations to reduce overfitting and handle high-cardinality categorical data. Its objective is similar to general gradient boosting:

12 $$L = \sum\limits_{{i = 1}}^{n} {l\left( {y_{i} ,\hat{y}_{i} } \right) + \lambda \sum\limits_{{j = 1}}^{p} {\Omega (T_{i} )} }$$

CatBoost ensures faster training, robustness, and improved missing or categorical data handling. These features make it ideal for recommendation systems, retail forecasting, and financial modeling. Cha et al. introduced CatBoost, outlining its advantages in efficiently handling categorical and tabular data^67,76–78^.

## Data analysis and explication

Polymer resin and biochar are designated as the key adsorbents in this investigation, and representative raw data of high quality are curated to build a reliable predictive model. The laboratory data are drawn from the same sources as those in former research works^14^. The dataset identified 1750 data points pertinent to adsorption isotherms featuring corresponding adsorbent properties. These points represent the adsorption of 73 organic materials on 50 biochar samples and 30 polymer resins. The input parameters are defined by eight factors: five Abraham descriptors (charge transfer, dipole-dipole interactions, cavity formation, which is the solvent’s ability to accommodate the solute molecule, proton donors reflecting solute’s capability to accept protons in hydrogen bonds, and proton acceptors indicating solute’s capability to give protons in hydrogen bonds), total pore volume (Vt), specific surface area (BET), and logCe (equilibrium concentration). The output parameter is logKd, which measures the adsorption degree.

**Fig. 6:**
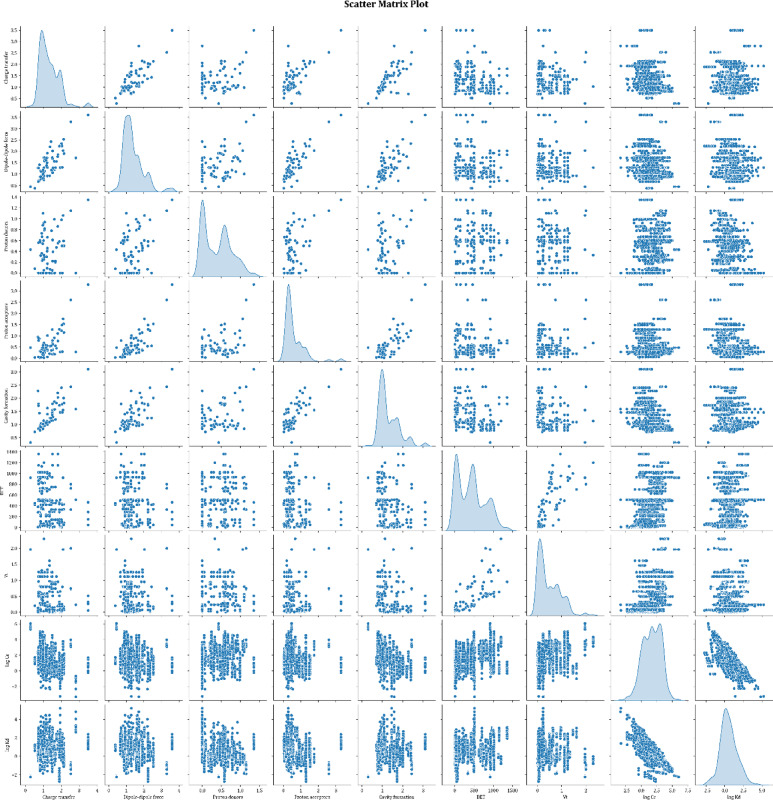
Scatter matrix plot illustrating variable relationships and distributions.

Figure 6 indicates the boxplot of all the considered variables in this study. Notice that from all the considered data points, 1225, 262, and 263 data points were considered for model training, testing, and validation, respectively.

The Pearson correlation coefficient measures the linear relationship between variables, varying from − 1 to + 1. A value of + 1 indicates a direct correlation, -1 signifies an inverse correlation, and 0 demonstrates a nonlinear relationship. The coefficient is determined through a formula that considers the variables of covariance and standard deviation. It is affected by the data’s linearity, normality, and outliers, which can influence its precision. The strength of the correlation is understood through the absolute value of the coefficient, where larger values signify stronger relationships. Statistical significance is frequently evaluated using hypothesis testing, where a p-value below 0.05 implies a noteworthy correlation. Visual displays, like scatter plots, are frequently utilized to verify the intensity and orientation of the relationship^78,79^:

13 $$\:{r}_{j}=\frac{\sum\:_{i=1}^{n}({I}_{i.j}-\stackrel{-}{{I}_{j}})({Z}_{i}-\stackrel{-}{Z})}{\sqrt{\sum\:_{i=1}^{n}{({I}_{i.j}-\stackrel{-}{{I}_{j}})}^{2}\sum\:_{i=1}^{n}{({Z}_{i}-\stackrel{-}{Z})}^{2}}}$$

**Fig. 7:**
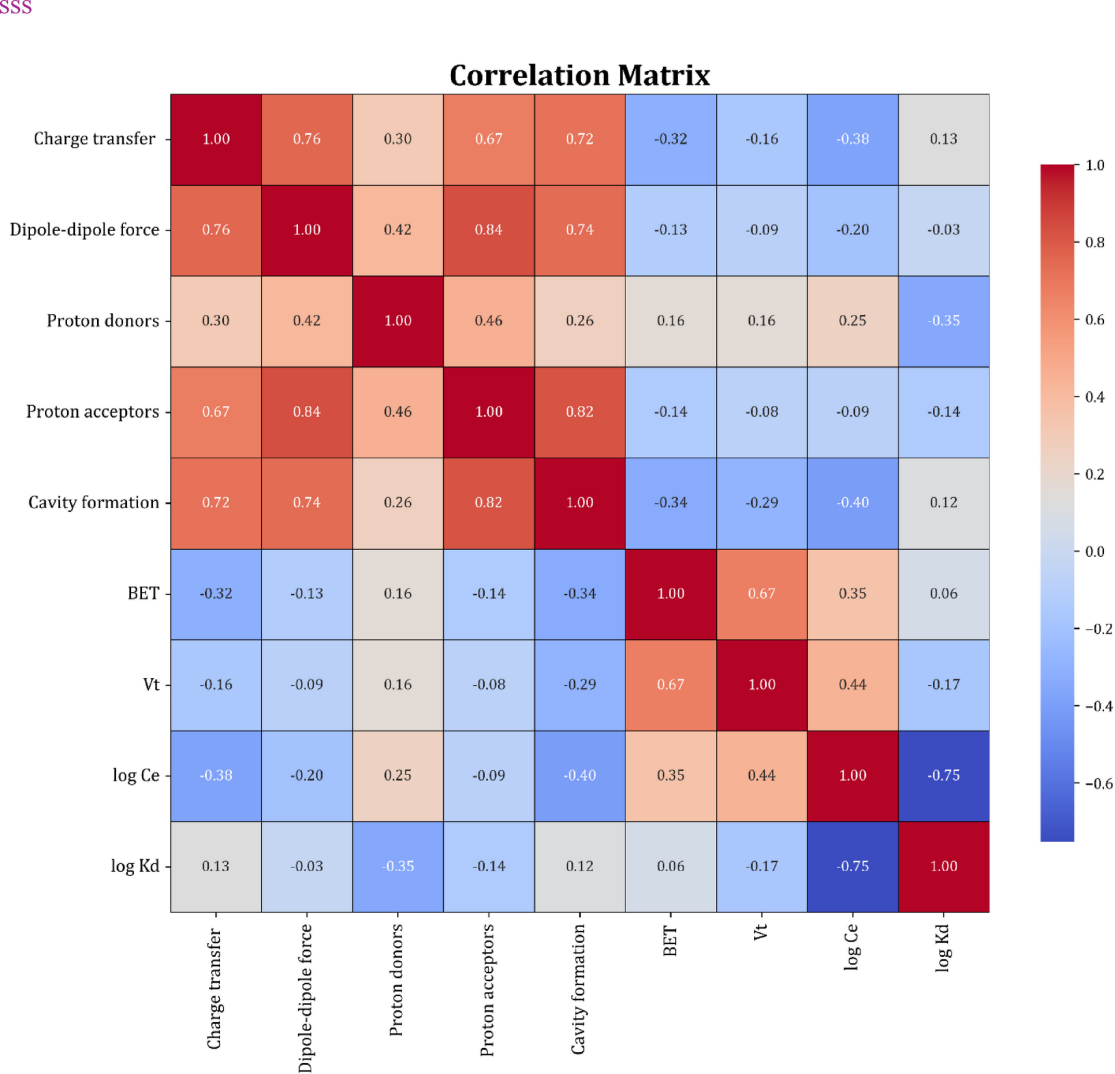
Correlation heatmap showing interrelationships among variable pairs analyzed in the study.

In this context, Z represents the second variable, indicated by its average value, Z̄. Additionally, Ī signifies the mean value of the approximate variable Ij. The connection between the input variables and the target factor being studied, particularly the adsorption of organic materials onto resin and biochar, reveals an intriguing dynamic. As illustrated in Fig. 7, it can be deduced that every input variable relates to the resin and biochar adsorption levels through five Abraham descriptors (charge transfer, dipole-dipole interactions, cavity formation, which pertains to the solvent’s capacity to hold the solute molecule, proton donors indicating solute’s ability to accept protons in hydrogen bonds, and proton acceptors denoting solute’s ability to release protons in hydrogen bonds), total pore volume (Vt), specific surface area (BET), and logCe (equilibrium concentration). Among these factors, the degree of adsorption is directly and positively linked to charge transfer, the specific surface area of the adsorbent, and the creation of cavities.

Before building machine learning models from data, assessing the dataset’s quality is crucial to identifying and resolving outliers. This study utilized the Monte Carlo Outlier Detection (MCOD) technique, a method well-regarded for its effectiveness and suitability in managing extensive and high-dimensional datasets. MCOD combines the principles of Monte Carlo sampling with density-based analytical techniques to identify outliers. Specifically, it detects anomalies by assessing the local density of data points within a representative subset of the dataset, enabling efficient recognition of those points that deviate substantially from their neighbors in terms of density. In this context, outliers are defined as data points significantly differing from others in their immediate neighborhood based on density criteria.

The MCOD method first selects a subset of the dataset using Monte Carlo sampling, where the subset is statistically representative of the entire data distribution. Density-based methods are then applied to analyze this subset and pinpoint outliers, dramatically reducing computational complexity compared to an exhaustive evaluation of the entire dataset. This makes MCOD fast and scalable, particularly suitable for high-dimensional and large-scale datasets like those used in this study. Despite its computational efficiency, MCOD does have a trade-off: the accuracy of outlier detection can vary depending on the sample size and the parameterization of key factors like the number of nearest neighbors (k). Proper calibration of these parameters is essential to maintain the robustness and reliability of the results. Nevertheless, MCOD is widely recognized as a practical solution for exploratory data analysis, particularly for applications where approximate results are sufficient, computational resources are limited, or real-time processing is required.

**Fig. 8:**
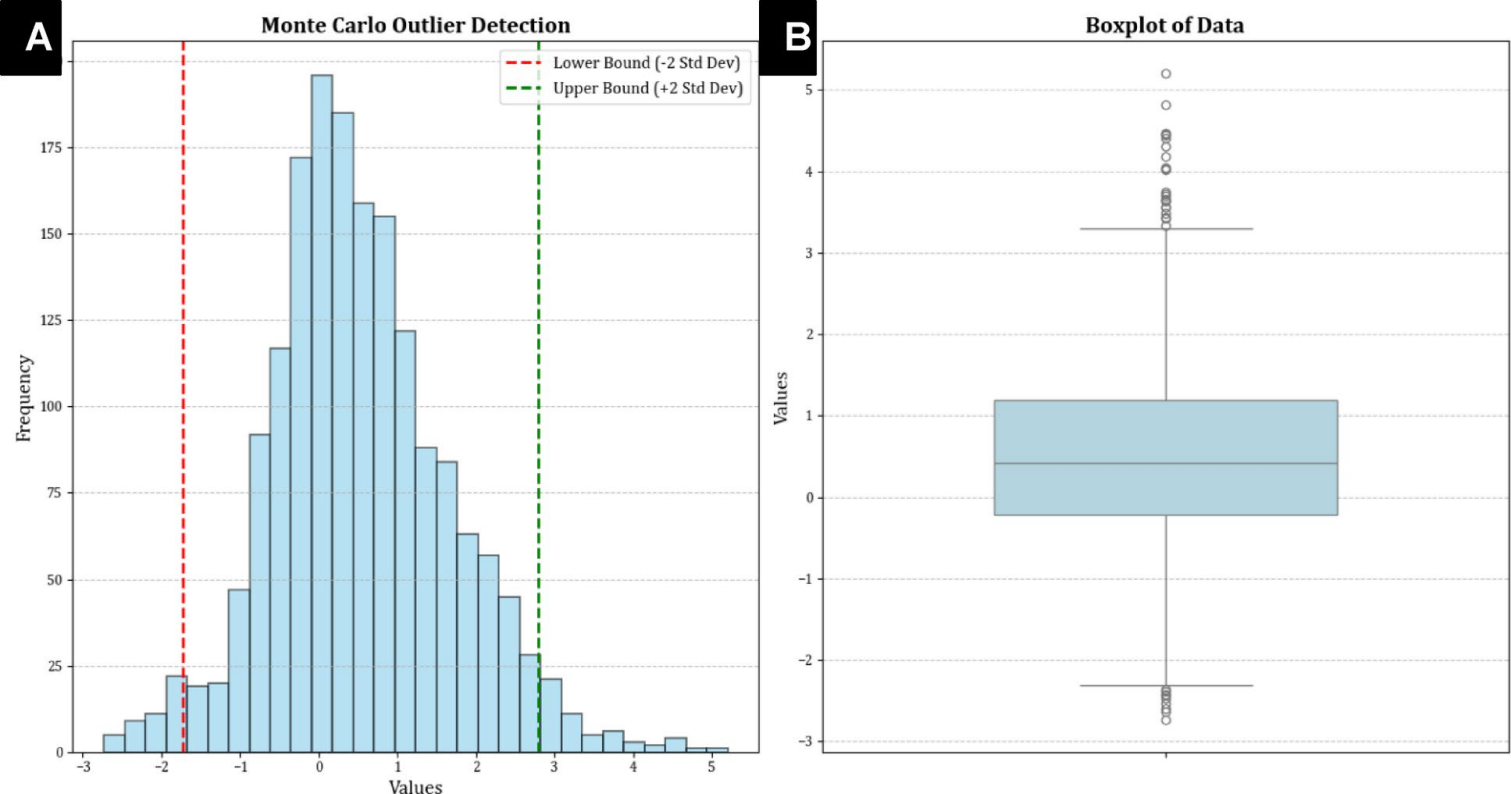
(**A**) Outlier detection using the Monte Carlo algorithm; (**B**) Boxplot representing data dispersion.

Using MCOD, this study ensures the dataset’s integrity while avoiding undue computational costs. The effectiveness of the technique in managing extensive datasets is demonstrated in Fig. 8, which provides a boxplot of the dataset used in this study. The boxplot visually depicts the dataset’s quality, highlighting that most data points fall within an appropriate range deemed suitable for model development. Using the entire cleaned dataset for model training ensures robust learning while enabling the machine learning models to generalize better to new and unseen data. By addressing overfitting and capturing both patterns and variations across the entire data range, the approaches adopted in this study deliver higher accuracy and reliability in predictive modeling for adsorption processes^80,81^.

## Results and discussions

This study utilizes machine learning methods to forecast the adsorption of organic materials onto resin and biochar. The research includes conventional techniques such as linear regression decision trees, sophisticated methods like convolutional neural networks (CNNs), and ensemble algorithms comprising XGBoost, LightGBM, and CatBoost. These various algorithms facilitate an in-depth examination of the most effective approaches for modeling intricate datasets, catering to nonlinear associations and interactions. Ensemble methods balance predictive accuracy and computational efficiency, making them ideal for large-scale applications requiring real-time predictions.

Essential evaluation metrics like the coefficient of determination (R²), residual standard deviation, mean squared error (MSE), and mean relative deviation percentage (MRD%) are used to evaluate model effectiveness. R² reflects the model’s ability to explain the variance in adsorption data, while MSE focuses on the accuracy of predictions. MRD% provides a scale-independent comprehension of error size. These metrics enable a detailed comparison between models, guaranteeing that the selected methods accurately reflect the fundamental processes of adsorption of organic materials onto resin and biochar^2,82^

14 $$\:R-squared\left({R}^{2}\right)=1-\frac{{\sum\:}_{i=1}^{N}{({\text{y}}_{\text{i}}^{\text{r}\text{e}\text{a}\text{l}}-{\text{y}}_{\text{i}}^{\text{p}\text{r}\text{e}\text{d}\text{i}\text{c}\text{t}\text{e}\text{d}})}^{2}}{{\sum\:}_{i=1}^{N}{({\text{y}}_{\text{i}}^{\text{r}\text{e}\text{a}\text{l}}-\overline{{\text{y}}^{\text{r}\text{e}\text{a}\text{l}}})}^{2}}$$

15 $$\:Mean\:squared\:error\:\left(MSE\right)\:=\:\frac{1}{N}{\sum\:}_{i=1}^{N}{({y}_{i}^{real}-{y}_{i}^{predicted})}^{2}$$

16 $$\:Residuals\:Standard\:Deviation\:\left(\sigma\:\right)=\sqrt{\frac{1}{N}{\sum\:}_{i=1}^{N}{({\text{y}}_{\text{i}}^{\text{r}\text{e}\text{a}\text{l}}-{\text{y}}_{\text{i}}^{predicted})}^{2}}$$

17 $$\:Mean\:relative\:deviation\:\left(MRD\right)=\frac{100}{N}{\sum\:}_{i=1}^{N}\left(\frac{{y}_{i}^{real}-{y}_{i}^{predicted}}{{y}_{i}^{real}}\right)$$

Here, $\:{\varvec{y}}_{\varvec{i}}^{\varvec{p}\varvec{r}\varvec{e}\varvec{d}\varvec{i}\varvec{c}\varvec{t}\varvec{e}\varvec{d}}$ and $\:{\varvec{y}}_{\varvec{i}}^{\varvec{r}\varvec{e}\varvec{a}\varvec{l}}$ denote the output and real target, respectively. N refers to the data points’ number in the dataset. The tuned hyperparameters of each algorithm is also tabulated in Table 1.

**Table 1:** Tuned structure for each algorithm.

Model	Tuned structure
Linear regression	None
Ridge regression	alpha: 4.281332398719396 (between 0.0001 and 10)
Lasso regression	alpha: 0.0001 (between 0.0001 and 10)
Elastic net	alpha: 0.004832930238571752, l1_ratio: 0.1 (0.0001 < alpha < 10 and 0 < l1_ratio < 1
SVR	C: 12 (between 1 and 100)
Random forest	Max_depth: 21 (between 1 and 100)
Gradient boosting	Max_depth: 6 (between 1 and 100)
KNN	K: 2 (between 1 and 50)
Decision tree	Max_depth: 21 (between 1 and 100)
XGBoost	Max_depth: 17 (between 1 and 100)
LightGBM	Max_depth: 31 (between 1 and 100)
CatBoost	Learning_rate: 0.023 (between 0.001 and 1)
Gaussian process	RBF kernel ( l: 0.14 and Sigma: 42.45, both between 1e-3 and 1e3)
ANN	Number of layers: 3; Number of neurons: 128, 64, 1; Transfer functions: ReLU (hidden), Linear (output)
CNN	Number of layers: 2 convolutional layers + 2 fully connected layers; Number of neurons: 16 filters, 32 filters, 64 neurons, 1 output neuron; Transfer functions: ReLU (convolutional), ReLU (fully connected), Linear (output)

**Fig. 9:**
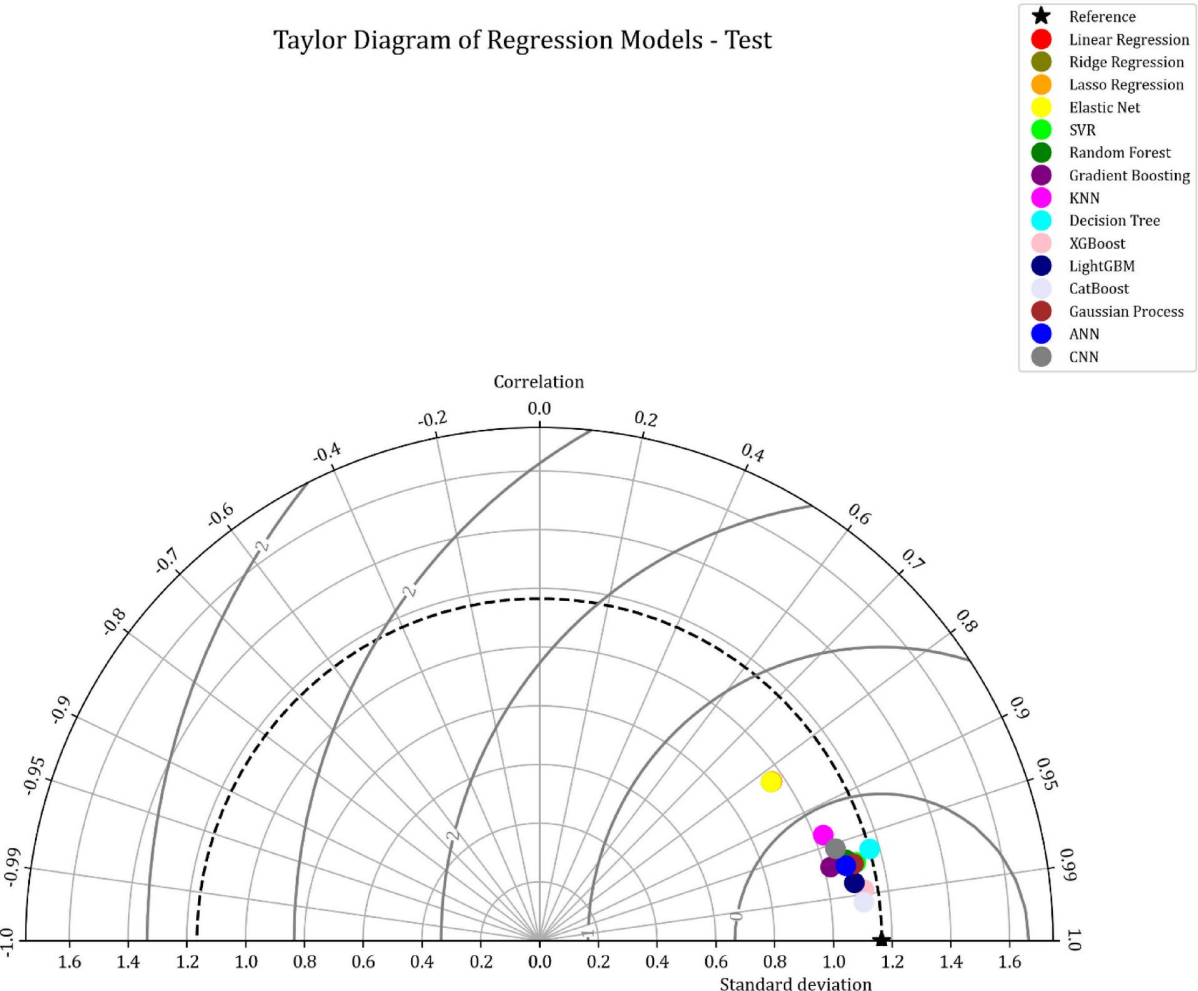

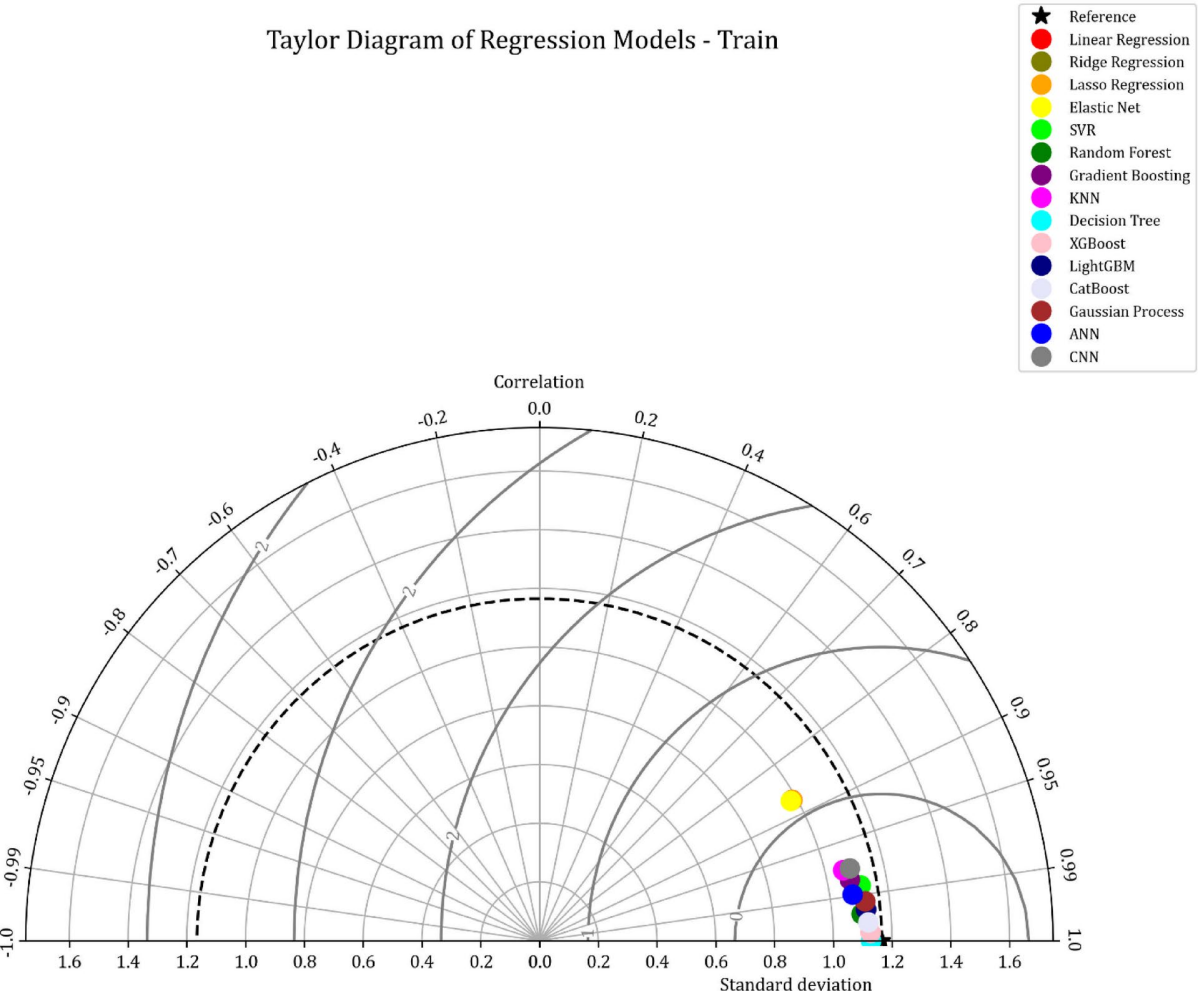

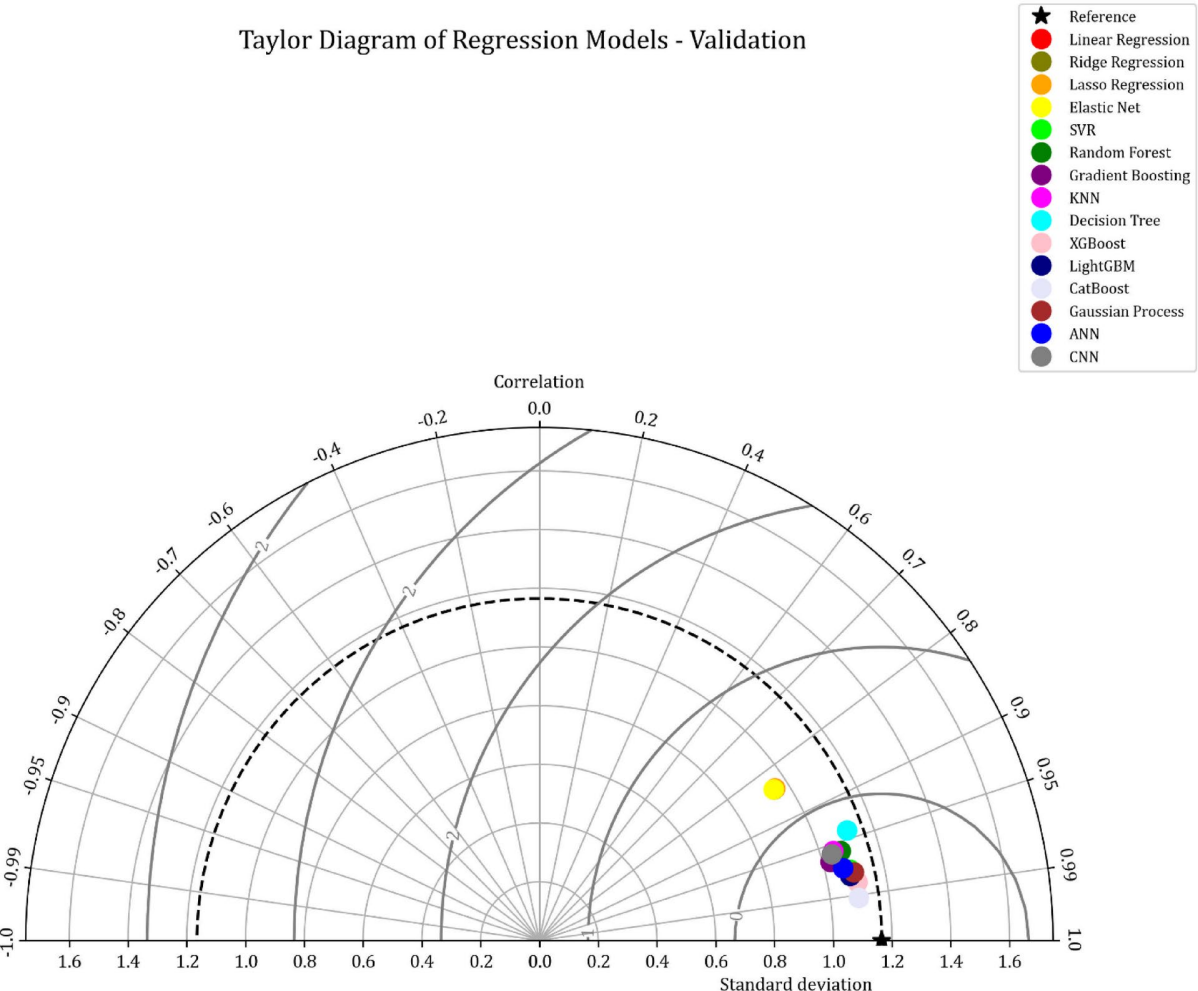
Taylor diagram comparing regression models based on correlation and standard deviation.

This study presents a comparative analysis of various machine learning regression models applied to a benchmark dataset. A Taylor Diagram (Fig. 9) is utilized to evaluate model performance by simultaneously displaying key metrics such as the correlation coefficient and standard deviation. This diagram facilitates a comparison of models, including Linear Regression, Ridge Regression, Lasso Regression, SVR, Random Forest, Gradient Boosting, KNN, Decision Trees, XGBoost, LightGBM, CatBoost, Gaussian Processes, ANN, and CNN. The proximity of each model’s point to the reference point in the diagram indicates its accuracy and alignment with the actual data, making it an effective tool for evaluating model performance.

The detailed analysis of the Taylor Diagram, supported by the data in Table 2, highlights XGBoost, lightGBM, and CatBoost as the top-performing models. These models exhibit strong correlations with the reference data and minimal standard deviation, evidenced by their proximity to the reference point. Table 2 shows that XGBoost achieves an R² value of 0.974 and an MSE of 0.0343, ligtGBM has an R^2^ of 0.964 and MSE of 0.0848, while CatBoost has an R² of 0.984 and an MSE of 0.0264. These results demonstrate the superior predictive accuracy of XGBoost, lightGBM, and CatBoost, outperforming the simpler models in terms of both R² and MSE.

**Table 2:** Developed evaluation metrics for every machine learning method used in this research by utilizing training, validation, and testing data portions.

Model	Train *R*²	Validation *R*²	Test *R*²	Train MSE	Validation MSE	Test MSE	Train MRD%	Validation MRD%	Test MRD%
Linear Regression	0.7622	0.6990	0.678	0.3027	0.3680	0.4364	312.83	199.01	279.46
Ridge Regression	0.7622	0.6990	0.678	0.3027	0.3679	0.4366	486.75	194.37	252.12
Lasso Regression	0.7622	0.6989	0.678	0.3027	0.3680	0.4364	315.39	198.50	268.99
Elastic Net	0.7622	0.6988	0.678	0.3027	0.3681	0.4367	618.87	192.70	270.46
SVR	0.9713	0.9507	0.941	0.0366	0.0602	0.0792	83.07	42.84	76.10
Random Forest	0.9926	0.9178	0.932	0.0094	0.1005	0.0916	26.23	59.18	69.57
Gradient Boosting	0.9616	0.9290	0.931	0.0489	0.0868	0.0935	91.45	184.55	118.51
KNN	0.9473	0.9147	0.875	0.0671	0.1042	0.1698	104.57	67.33	105.31
Decision Tree	1.0000	0.8804	0.926	0.0000	0.1462	0.0996	0.00	126.46	64.70
XGBoost	0.9994	0.9669	0.974	0.0008	0.0404	0.0343	11.37	71.03	70.19
LightGBM	0.9907	0.9579	0.964	0.0118	0.0514	0.0484	28.77	219.01	106.88
CatBoost	0.9968	0.9823	0.984	0.0041	0.0216	0.0212	20.93	18.41	44.54
Gaussian Process	0.9854	0.9545	0.942	0.0186	0.0556	0.0779	19.30	295.48	43.57
ANN	0.9766	0.9453	0.940	0.0297	0.0669	0.0808	61.80	42.03	93.95
CNN	0.9419	0.9048	0.897	0.0739	0.1164	0.1395	97.30	46.96	88.10

In contrast, simpler models such as Linear Regression, Ridge Regression, Lasso Regression, Elastic Net, Decision Trees, and KNN show significantly lower performance. These models have reduced R² values, ranging from 0.678 to 0.875, and higher MSE values, ranging from 0.18 to 0.43. These results align with existing research, which consistently finds that advanced models like XGBoost, lighGBM, and CatBoost generally outperform more straightforward regression tasks^77,79,83^. This study emphasizes the importance of choosing more complex models for datasets that require high accuracy and are characterized by complex relationships.

This research employs graphical methods, including relative deviation values and cross plots, to assess the effectiveness and dependability of different machine learning algorithms in forecasting the adsorption levels of biochars and resins. These visual aids are essential for offering thorough insights into model performance by emphasizing patterns and differences between expected and actual values.

**Fig. 10:**
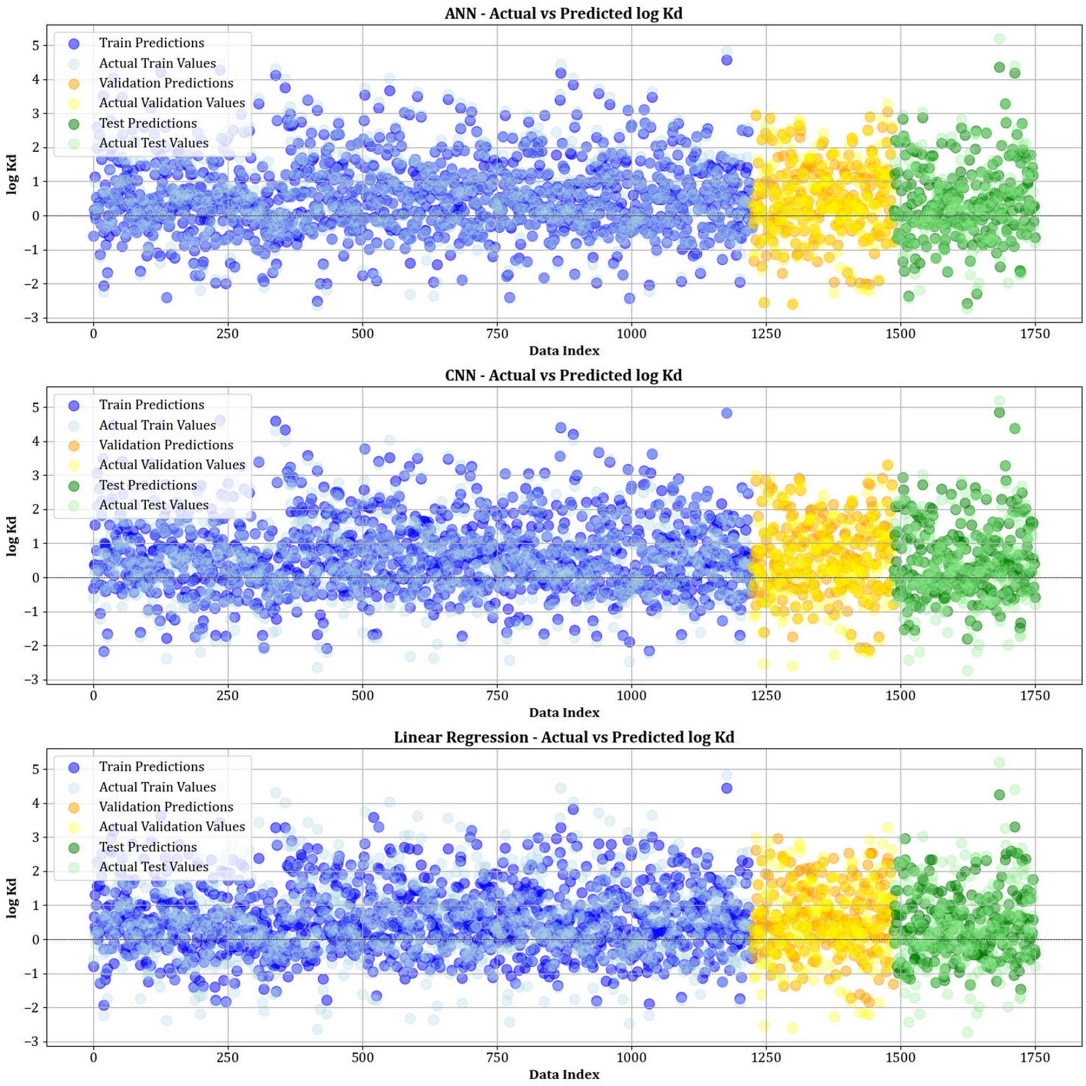

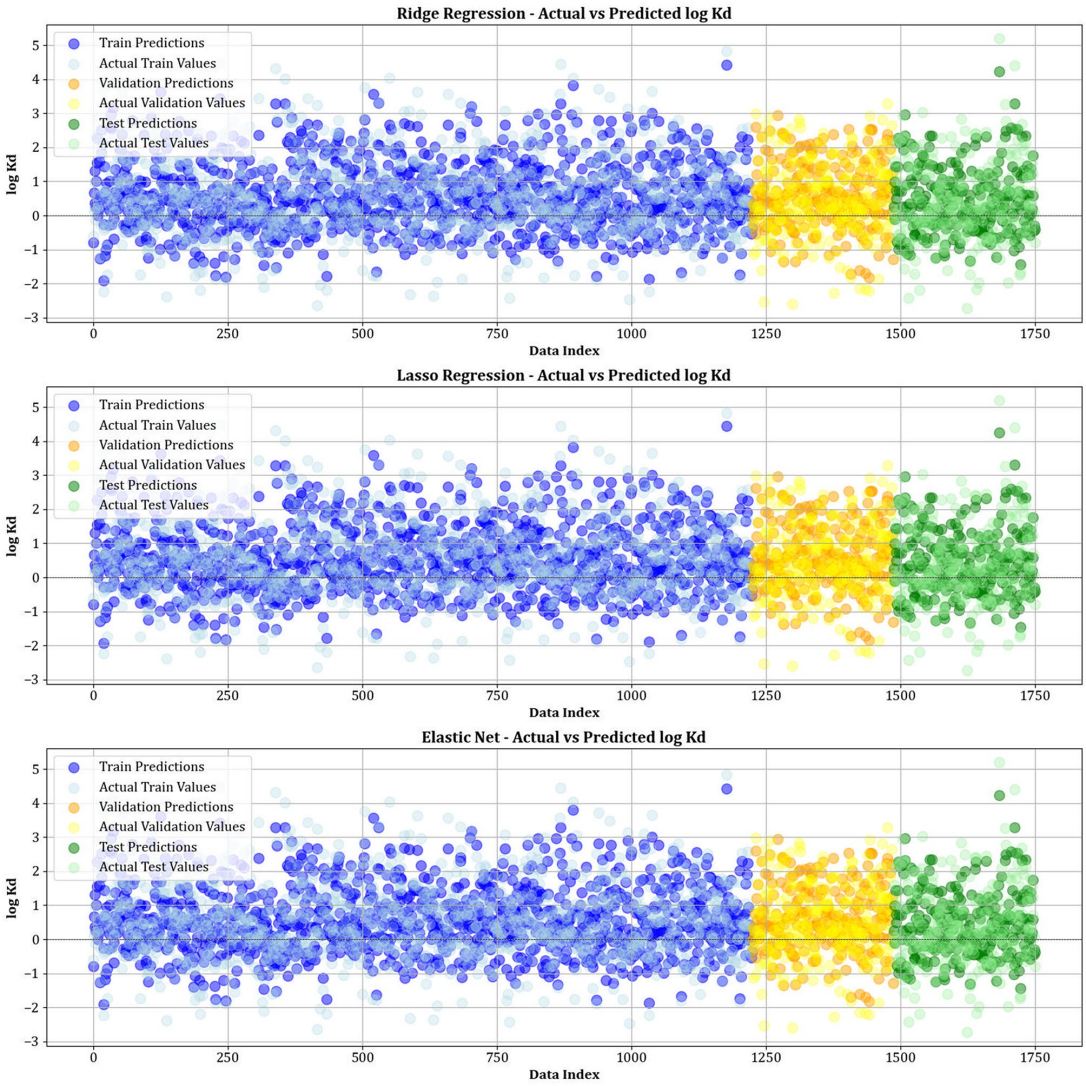

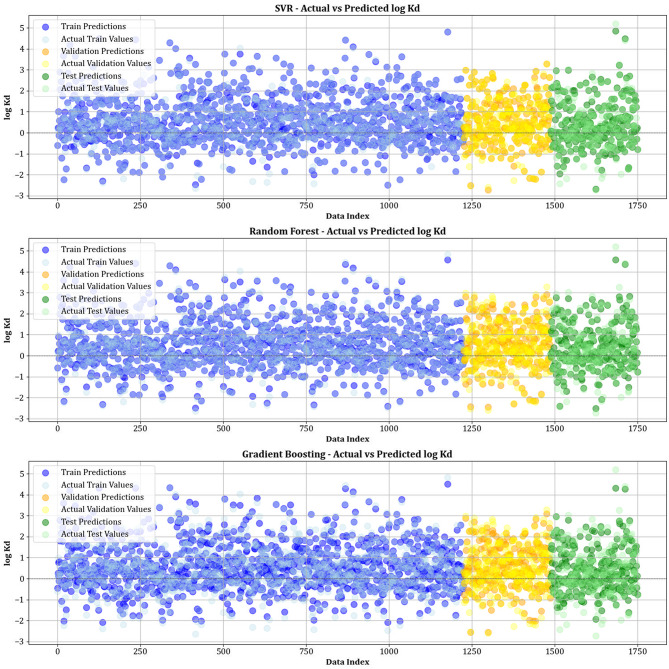

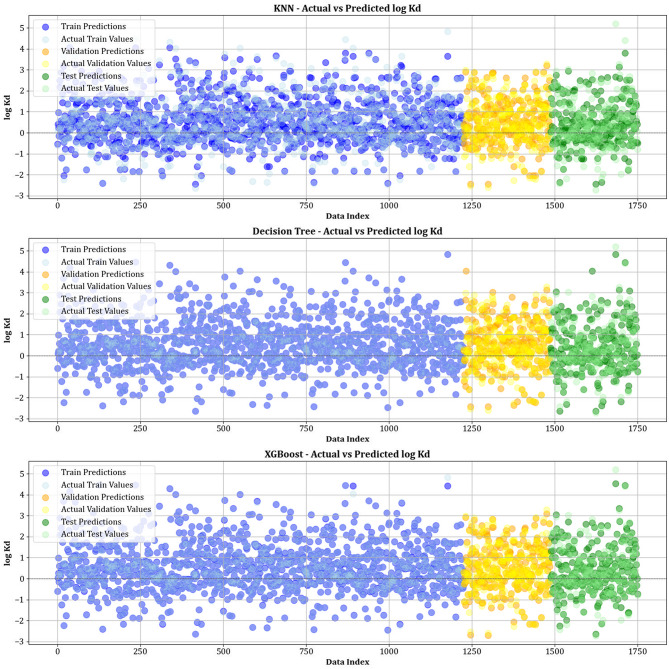

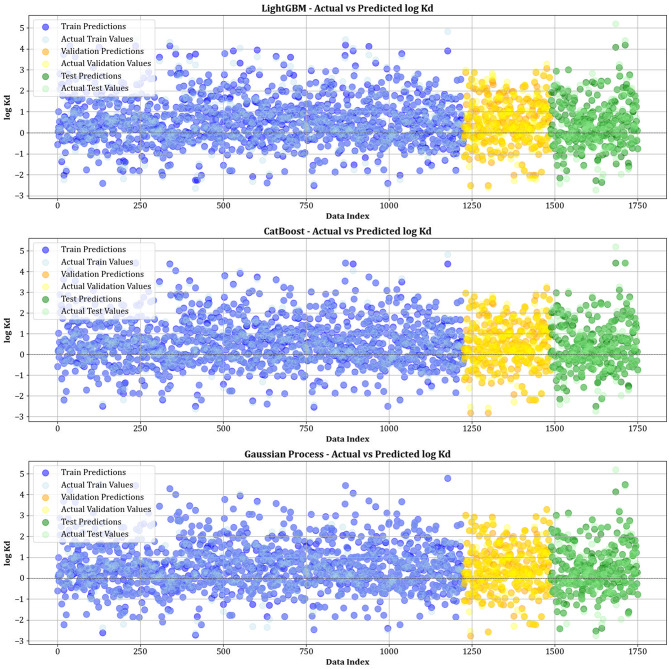
Comparison of actual vs. predicted values over training, testing, and validation datasets for all models.

**Fig. 11:**
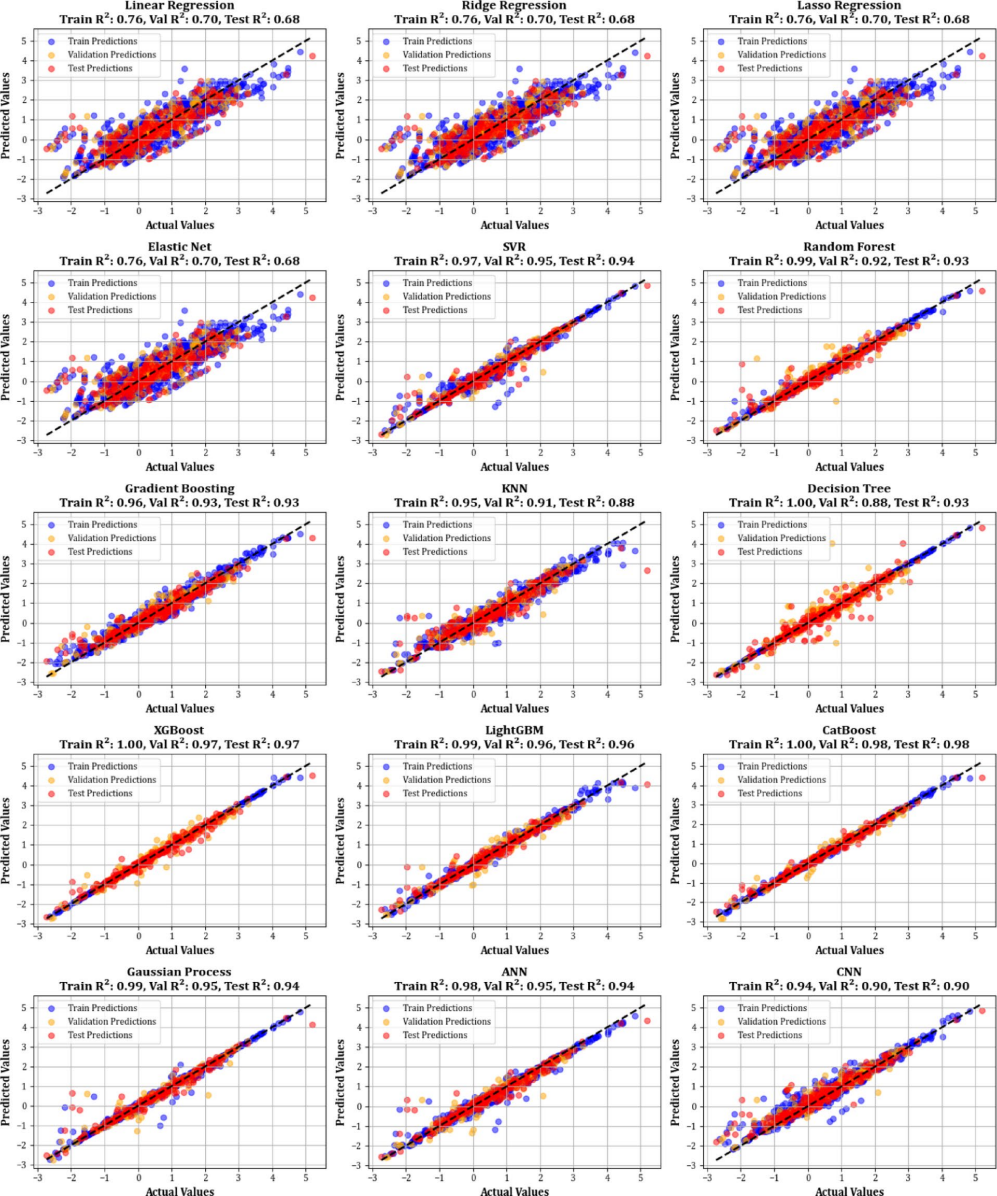
Cross plots of modeled versus actual values for training, testing, and all datasets across different models.

Figure 10 contrasts actual and simulated data points from various datasets, encompassing training, testing, and validation sets for all the models created. The analysis shows that the XGBoost, lightGBM, and CatBoost algorithms demonstrate almost the exact alignment between actual and predicted values, highlighting their modeling capabilities. This strong alignment reflects their remarkable ability to capture complex relationships in the dataset. Moreover, Fig. 11 displays cross-validation graphs showing the relationships between actual and predicted values for all machine learning models. Interestingly, in the XGBoost, LightGBM, and CatBoost models, data points broadly group around the bisector line (y = x), and their fitted lines closely follow this bisector. This alignment confirms their strong predictive ability, supporting their skill in correctly predicting results that correspond with actual observations.

**Fig. 12:**
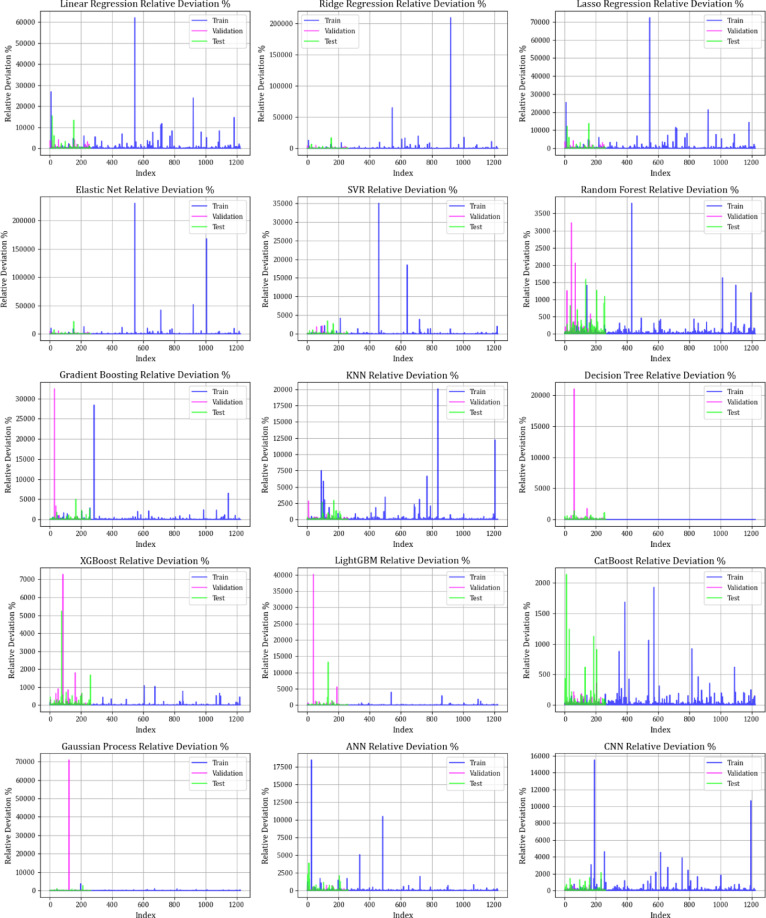
Relative percentage deviation for training, testing, and validation datasets for all models.

**Fig. 13:**
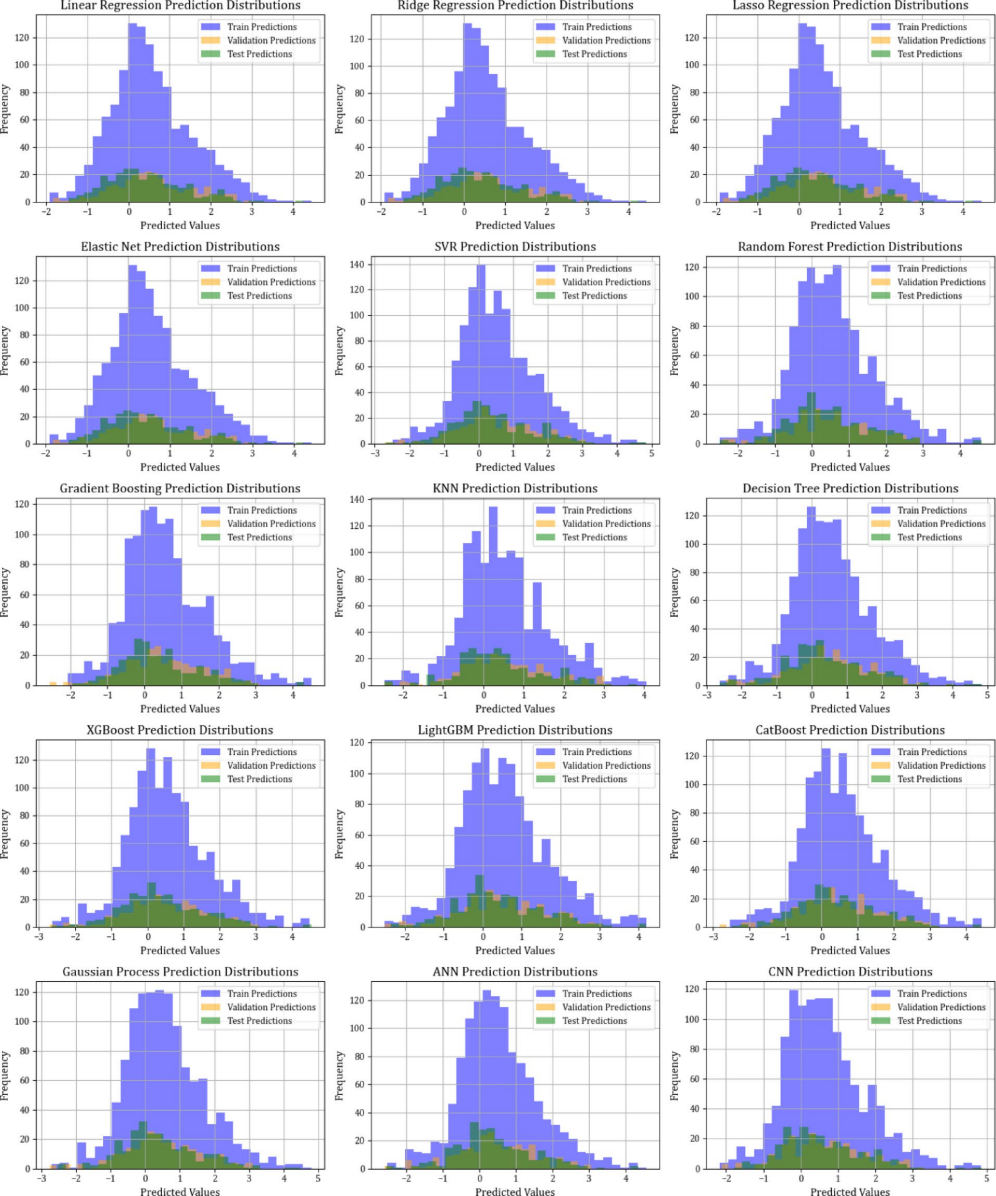
Data distribution (counts) among training, testing, and validation datasets for all analyzed models.

Figure 12 examines error distributions via scatter plots of relative errors. The errors for the XGBoost, LightGBM, and CatBoost models are evenly distributed around the x-axis, indicating low variance and steady predictive performance. This assessment further strengthens the trustworthiness of these models in reducing prediction errors, thus boosting confidence in their forecasting abilities. Figure 13 demonstrates the models’ predictive distribution abilities throughout the training, testing, and validation stages. The prediction frequency of the XGBoost, lightGBM, and CatBoost models exhibits more consistency across these segments than other algorithms, highlighting their resilience and reliability. This uniformity across various modeling phases underscores these models as the most reliable for practical applications in the adsorption of organic materials onto resin and biochar.

The graphical analysis demonstrates that XGBoost, lightGBM, and CatBoost models efficiently predict the adsorption of organic materials onto resin and biochar, exhibiting low error and consistent performance across different datasets. These results indicate their appropriateness for real-world uses where precise forecasting is essential.

Assessing the significance of features is crucial for understanding how input variables contribute to predicting the target variable in machine learning applications. In this study, we utilize the SHAP (Shapley Additive exPlanations) method to explore the complex relationships between features and their significance. From game-theoretic principles, SHAP offers a robust framework for interpreting model outputs, facilitating a detailed understanding of each feature’s contribution to the model’s predictions.

**Fig. 14:**
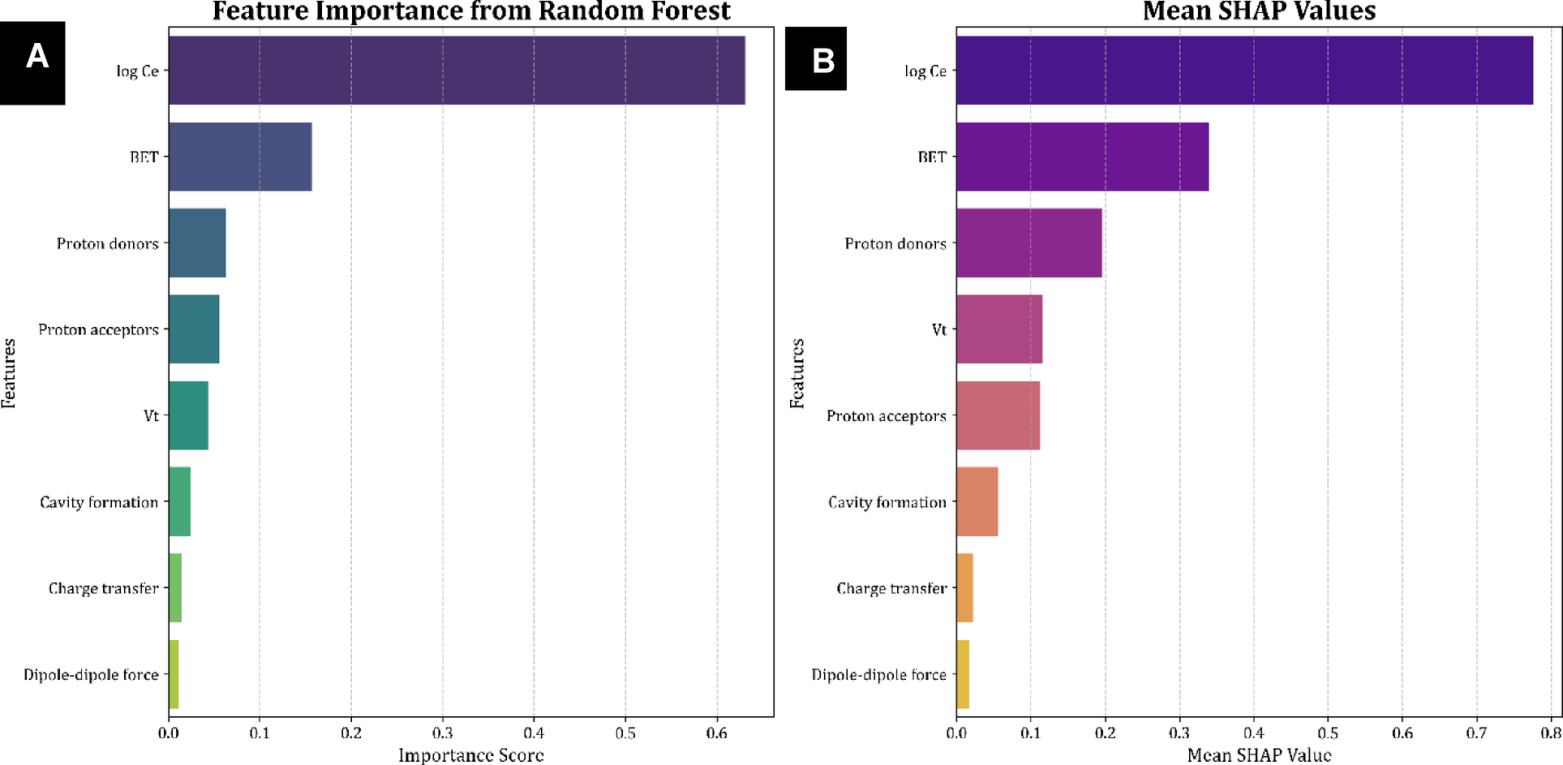
(**A**) Feature importance derived from the random forest model; (**B**) Mean SHAP values illustrating feature relevance.

Figure 14 illustrates the SHAP values associated with the input features and the feature importance derived from a Random Forest model used to predict the adsorption of organic materials onto resin and biochar. The features are ranked in descending order based on their average SHAP values, with those ranked highest exerting the most significant impact on the model’s outcomes. This analysis indicates that equilibrium concentration and particles’ specific surface area are the primary determinants of the adsorption degree of resins and biochars. These findings are critical for identifying the key factors governing resins and biochar adsorption degree, providing valuable insights for future research and optimization strategies. This clarity enhances model performance and applicability in real-world scenarios, ultimately allowing stakeholders to make data-driven decisions by understanding the unique influence of each feature on the model’s predictions.

## Future scope

The present study provides a robust framework for modeling the adsorption of organic compounds onto biochars and resins using data-driven machine-learning techniques. However, there are several opportunities for extending and improving this work. First, additional experimental datasets that consider broader variations in biochar and resin types and environmental conditions such as temperature, pH, and pressure could enhance the generalizability of the models. Second, future research can focus on hybrid models integrating physics-based adsorption equations with machine-learning techniques to balance predictive accuracy and interpretability. Third, studies could explore scalability by adapting the models to handle larger datasets from industrial applications or real-time monitoring systems. Another promising direction is applying transfer learning methods, which allow trained models to adapt to adsorption scenarios with minimal additional data. Finally, incorporating domain-specific optimization techniques and exploring other advanced interpretability frameworks (beyond SHAP analysis) can refine the insights into adsorption mechanisms and improve practical applicability. These future developments will ensure the continued improvement of predictive modeling in adsorption processes while broadening its scope in environmental sustainability research.

## Conclusion

This research focuses on developing various data-driven models using machine learning algorithms to predict the adsorption of organic compounds on resins and biochars. The models leveraged key descriptors such as Abraham descriptors (charge transfer, dipole-dipole interactions, cavity formation, proton donors, proton acceptors), total pore volume (Vt), specific surface area (BET), and equilibrium concentration (logCe). Various metrics and visualizations were employed to evaluate their performance, while the Monte Carlo technique ensured the dataset’s integrity by efficiently handling outliers. Among these models, XGBoost, LightGBM, and CatBoost emerged as the best-performing techniques, consistently demonstrating high R² values and low error metrics across training, validation, and testing datasets. A sensitivity analysis revealed a strong direct correlation between charge transfer, cavity formation, and adsorption characteristics, highlighting their significance in predicting adsorption levels. Furthermore, SHAP analysis identified equilibrium concentration and specific surface area as the most influential features in determining adsorption behavior. These insights improve the model’s interpretability and reliability, offering a strong foundation for understanding adsorption-related mechanisms. However, this study has certain limitations, including its reliance on adsorption data restricted to specific biochars and resins and excluding external factors (e.g., temperature, pH) that may influence adsorption. Additionally, while the focus here was on high-dimensional machine learning models, future studies could explore domain-specific hybrid models to enhance interpretability and address scalability challenges in real-world settings. This study underscores the superior performance of advanced data-driven models such as XGBoost, LightGBM, and CatBoost in capturing intricate relationships in adsorption-related data. Despite the identified limitations, these models’ predictive accuracy and interpretability emphasize their potential for practical applications, contributing significantly to adsorption process optimization and environmental sustainability efforts.

## Data Availability

The data that support the findings of this study are available from the corresponding author, Mohammad Reza Kezemi, upon reasonable request.
